# Metabolic Insights into the Anion-Anion Antagonism in Sweet Basil: Effects of Different Nitrate/Chloride Ratios in the Nutrient Solution

**DOI:** 10.3390/ijms21072482

**Published:** 2020-04-03

**Authors:** Giandomenico Corrado, Luigi Lucini, Begoña Miras-Moreno, Pasquale Chiaiese, Giuseppe Colla, Stefania De Pascale, Youssef Rouphael

**Affiliations:** 1 Department of Agricultural Sciences, University of Naples Federico II, 80055 Portici, Italy; chiaiese@unina.it (P.C.); depascal@unina.it (S.D.P.); youssef.rouphael@unina.it (Y.R.); 2 Department for Sustainable Food Process, Research Centre for Nutrigenomics and Proteomics, University Cattolica del Sacro Cuore, 29122 Piacenza, Italy; mariabegona.mirasmoreno@unicatt.it; 3 Council for Agricultural Research and Economics- Research Centre for Genomics and Bioinformatics (CREA-GB), via San Protaso 302, 29017 Fiorenzuola d’Arda, PC, Italy; 4 Department of Agriculture and Forest Sciences, University of Tuscia, 01100 Viterbo, Italy; giucolla@unitus.it

**Keywords:** *Ocimum basilicum*, metabolomics, nutrient solution, leaves, hydroponic, anions, stress response

## Abstract

Sweet basil (*Ocimum basilicum* L.) is a highly versatile and globally popular culinary herb, and a rich source of aromatic and bioactive compounds. Particularly for leafy vegetables, nutrient management allows a more efficient and sustainable improvement of crop yield and quality. In this work, we investigated the effects of balanced modulation of the concentration of two antagonist anions (nitrate and chlorine) in basil. Specifically, we evaluated the changes in yield and leaf metabolic profiles in response to four different NO_3_^−^:Cl^−^ ratios in two consecutive harvests, using a full factorial design. Our work indicated that the variation of the nitrate-chloride ratio exerts a large effect on both metabolomic profile and yield in basil, which cannot be fully explained only by an anion-anion antagonist outcome. The metabolomic reprogramming involved different biochemical classes of compounds, with distinctive traits as a function of the different nutrient ratios. Such changes involved not only a response to nutrients availability, but also to redox imbalance and oxidative stress. A network of signaling compounds, including NO and phytohormones, underlined the modeling of metabolomic signatures. Our work highlighted the potential and the magnitude of the effect of nutrient solution management in basil and provided an advancement towards understanding the metabolic response to anion antagonism in plants.

## 1. Introduction

Sweet basil (*Ocimum basilicum* L.) is an annual, herbaceous, aromatic species belonging to the Lamiaceae family. Basil is arguably the finest and most versatile culinary and aromatic herb [1,2]. This species has a global importance not only as a widely employed food garnish, but also as a raw material for phyto-chemical preparations with proven health benefits [3], as a folk medicine, and as an ornamental plant [4,5].

The quality of the commercial product is largely influenced by pre-harvest factors (e.g., genotype, growing conditions and agronomic practices) [6,7,8]. Moreover, the age of the leaves at harvest also determines quality, considering that in both professional and amateur production, leaves and stems are harvested at least twice per growing cycle. All these factors are expected to ultimately affect the quality of the edible product [9,10]. Like other culinary herbs, the commercial quality is defined by a large array of chemically diverse molecules that also influence the sensory profile [2]. It is well known that the basil is a source of a vast assortment of bioactive compounds, such as phenol derivatives, terpenoids, flavonoids and phenylpropanoids, with known biological, pharmaceutical and industrial uses [11,12,13]. 

Particularly for leafy vegetables, the precise nutrient solution (NS) management in soilless cultivation is a widely used, affordable and efficient way to boost yield and quality in professional horticulture [14]. For instance, a mild-to-moderate chemical stress (e.g., salt or nutrient stress) has proven to be useful to improve the sensory and functional quality of vegetables through the increased accumulation of stress-responsive chemicals [8,14]. Moreover, macronutrient management and, in particular, for leafy green vegetables, optimum nitrogen application is essential to balance the always valued increase of plant metabolism with a more limited accumulation of nitrates in edible organs. Among leafy vegetables, basil is second only to rocket (*Eruca vesicaria* (L.) Cav.) in terms of nitrate accumulation in leaves [15]. Controlling the concentration of nitrates in plant tissues is one of the problems of producing high quality vegetables (Reg. EC 194/97). 

Despite its global economic importance, little is known on the genomics and transcriptomics of sweet basil, as well as of other Lamiaceae. The complexity and variety of the compounds of interests in basil implies that a deeper understanding of its response to growing conditions can currently be achieved through an untargeted metabolomics approach. Under this perspective, basil is considered to be an emerging system to improve our understanding on the link between growing conditions and flavor compounds in plants [2]. 

In this study, we tested whether the concomitant modulation of nitrates and chlorine concentrations in the NS can induce broad responses in basil. In particular, our goal was to understand the changes in the metabolic profile of the leaves in response to four different NO_3_^−^:Cl^−^ ratios in two consecutive harvests, taking into account the effects on plant yield (e.g., stem and leaves).

## 2. Results

### 2.1. Plant Biomass

The results regarding plant biomass and mineral composition in relation to the tested factors (NO_3_^−^:Cl^−^ ratios [R] and cuts [CT]) are presented in Table 1. The main effect of decreasing NO_3_^−^:Cl^−^ ratios was a reduction of fresh shoot biomass. When averaged over the number of cuts, the total shoot biomass decreased linearly with increasing Cl^−^ amounts in the nutrient solution (NS) from 40 to 80. A significant difference was not recorded between the 80:20 and the 60:40 R. Regarding the effect of the number of cuts, the fresh shoot biomass of basil recorded 75 DAT (CT2), which declined by 16% compared to the plants harvested 47 DAT (CT1) (Table 1). The nitrate concentration of sweet basil was only affected by the NO_3_^−^:Cl^−^ ratio, whereas the chloride concentration was significantly influenced by both tested factors and their interaction (Table 1). When averaged over number of cuts, the nitrate concentration in basil leaves decreased linearly with increasing Cl^−^ ratio in the NS from 40 to 80, with no significant difference between the 80:20 and the 60:40 treatments (Table 1). Finally, the chloride concentration in basil leaves increased from the 80:20 to the 20:80 NO_3_^−^:Cl^−^ ratio; however the accumulation of chloride in the leaf tissue was more pronounced in the second harvest under severe chloride conditions (i.e., 20:80 R) (Table 1).

### 2.2. Effect of the Different NO_3_^−^:Cl^−^ Ratios on Nitrate Transporters’ Expression in Leaves

To verify possible responses to the different nutrient regimes related to N transport (e.g., xylem unloading and mesophyll import) in the target tissue, we analyzed the basil leaves of the second cut, mainly because of the lack of a significant main effect of the CT factor over nitrate accumulation.

#### 2.2.1. Identification of Putative Nitrate Transporters in Basil

Considering the lack of genomics information in *O. basilicum*, we performed a bioinformatics analysis to identity transcripts putatively coding for nitrate transporters. We first built a database of protein sequences that are related to the terms “nitrate trasporter” or “NRT1” in Viridiplante from the National Center for Biotechnology Information (NCBI) database, yielding a collection of 9270 unique protein sequences. We then retrieved from NCBI the available *O. basilicum* expressed sequences (23.845 ESTs and 85 cDNAs), with an average length of 634.8 ± 181.0 bp (Supplementary Figure S1). The nucleotide sequences of *O. basilicum* (query) were compared with the protein sequences of interest using the blastx algorithm. In total, 573 transcripts found a significant similarity (e-value cut-off: < 1E-10) within our database of non-redundant protein sequences. Many basil sequences (*n* = 468) were similar to a single protein sequence, but 15 sequences matched 20 or more proteins. The percentage of significant matches (2.4% of ESTs) should be gauged, considering that the EST libraries were not normalized. Redundancy was filtered out with the “best-blast-hit” method, considering the difficulty in establishing a threshold of similarity between ESTs that can define an identity between “entries”. After the removal of redundancy, we found 39 unique best-hits, all represented by ESTs. They were classified into four groups, namely: nitrate transporters (sensu stricto) with 16 unique hits corresponding to 64 ESTs; peptide transporters (e.g.,: ABC transporters) with 11 unique hits corresponding to 24 ESTs; transporters/channels of ions (mainly potassium) with six unique hits corresponding to 41 ESTs; and “others” (such as proteins indirectly linked to the transport of nitrates) with five unique hits corresponding to 433 ESTs), according to a manually curated phylogenetic analysis (see Material and Methods). Information on the 16 unique hits classified as nitrate transporters is reported in Supplementary Table S1.

#### 2.2.2. Gene Expression Analysis

**Figure 1:**
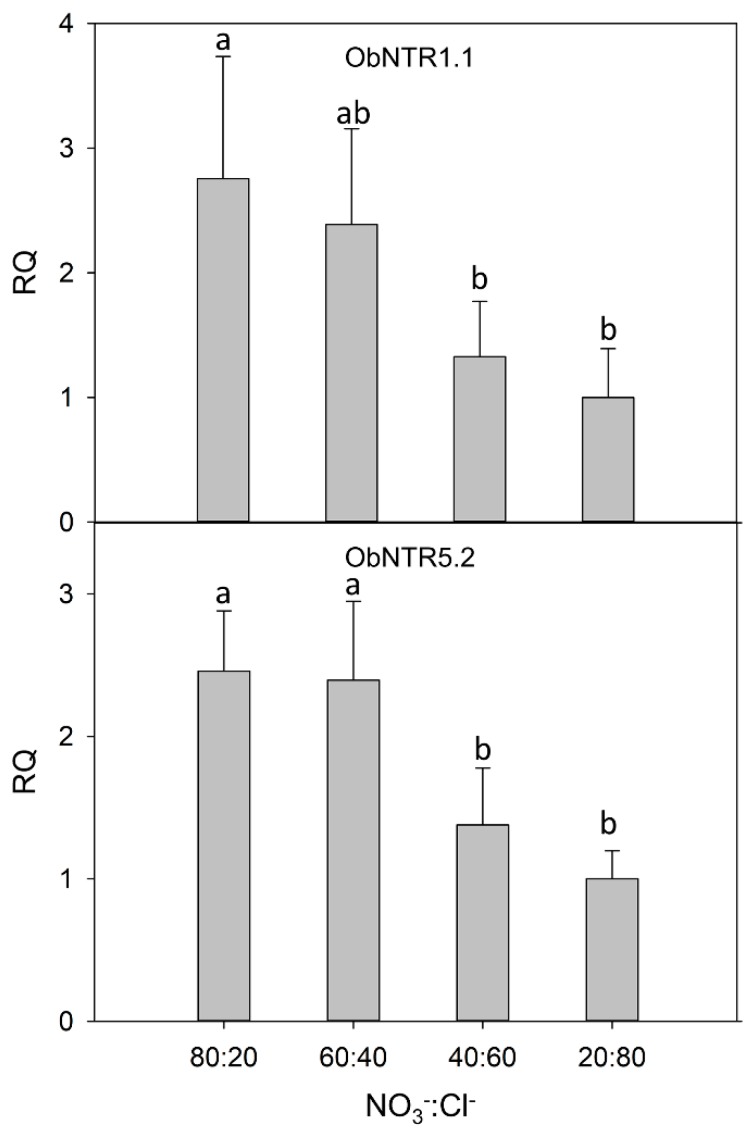
Relative gene expression by real-time RT-PCR of the putative nitrate transporter ObNPF1.1 and ObNPF5.2. For each condition, the relative quantity (RQ) is shown with respect to the calibrator condition (20:80). Different letters (a and b) indicate that the 2^−ΔCt^ values are significantly different (*p* ≤ 0.05).

The level of expression of the two transcripts (ObNPF1.1 and ObNPF5.2; see Supplementary Table S2 for their naming according to [16]) was analyzed by real-time RT-PCR in leaves of plants under different nutritive R. The relative quantification was performed considering the lowest NO_3_^−^:Cl^−^ ratio (20:80), as calibrator condition. The Glyceraldehyde-3-phosphate dehydrogenase (GAPDH) gene was used as a reference gene. The results indicated that the analyzed transcripts are overexpressed in the leaves of the plants fed with the highest amount of nitrates (Figure 1). Moreover, the gene expression analysis indicated the lack of a significant difference between the 80:20 and 60:40 R.

### 2.3. Metabolomics of Basil Leaves

To elucidate the plant response to different NO_3_^−^:Cl^−^ ratios and cuts, the metabolic composition of basil leaves was evaluated using a high-resolution untargeted UHPLC-QTOF mass spectrometric approach. More than 1800 compounds were annotated based on the PlantCyc database. Their multivariate statistics allowed to define distinctive patterns and to identify phytochemical profiles that depend on the harvests and the NS composition.

**Figure 2:**
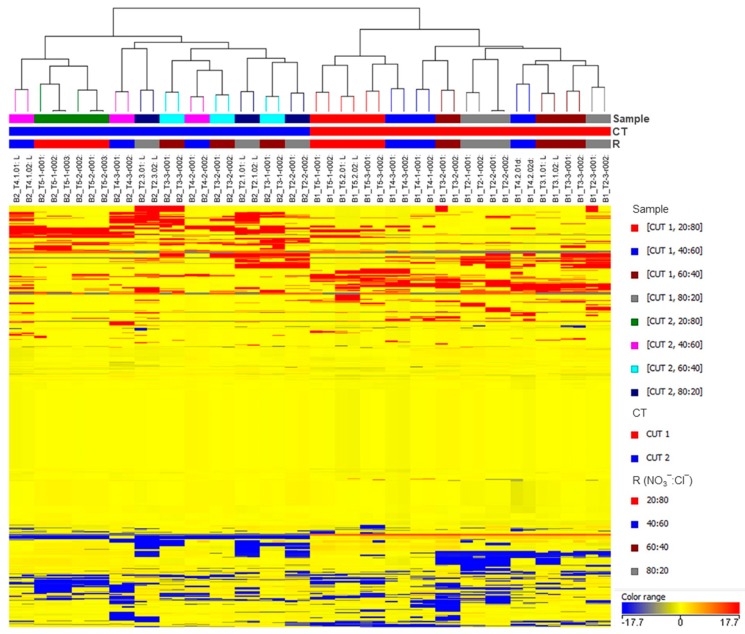
Unsupervised hierarchical clustering of the metabolic profile of basil leaves in the different experimental samples. Samples (i.e., each different biological replicate per condition) are identified by colored segments of the top-bars. Color codes are presented the right-hand side per factor. A fold-change based heatmap was built and samples were clustered with the Ward’s algorithm, based on Euclidean distances.

The unsupervised hierarchical cluster analysis (HCA) was performed using the Ward’s minimum variance method on the pairwise dissimilarity between samples. With this aim, a heat-map based on fold-change values was used and Euclidean distance was adopted. Samples were clustered according to the CT factor in two experimentally coherent groups, CT1 and CT2 (Figure 2), suggesting that harvesting leaves at different cuts has a leading impact on the metabolome’s variance in basil.

**Figure 3:**
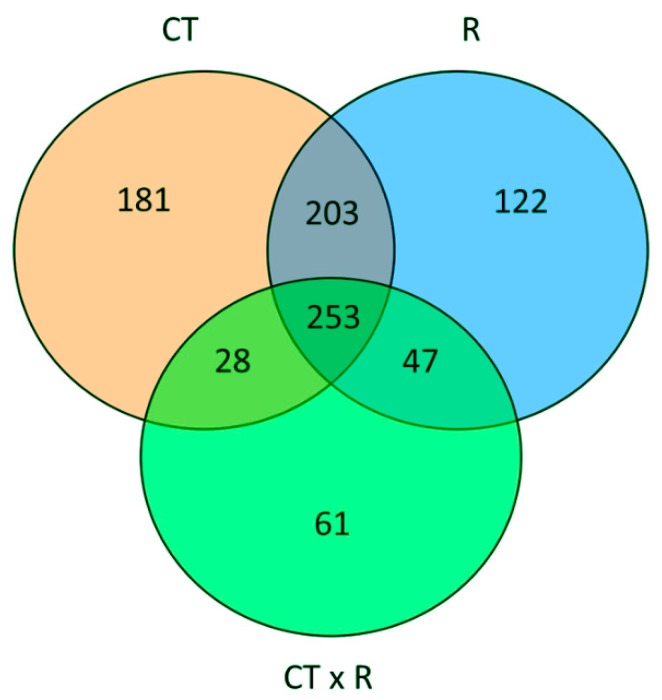
Venn diagram summarizing the result of two-way independent ANOVA of the metabolic profiles, considering as factor the NO_3_^−^: Cl^−^ ratio (R) and cut (CT). The graph displays the number of unique and overlapping metabolites that accumulate differentially for each factor and their interaction (significance: *p* < 0.05 with FDR correction).

To quantify the main effect of the factors under investigation and their interaction, we performed a two-way analysis of variance of the metabolic profiles. This analysis indicated that the CT had the largest effects in term of differentially accumulated metabolites, followed by the R. Moreover, the interaction between the factors also accounts for an almost similar number of statistically different metabolites, indicating that the basil response to the different nutrient ratios is also considerably affected by the CT (Figure 3).

**Figure 4:**
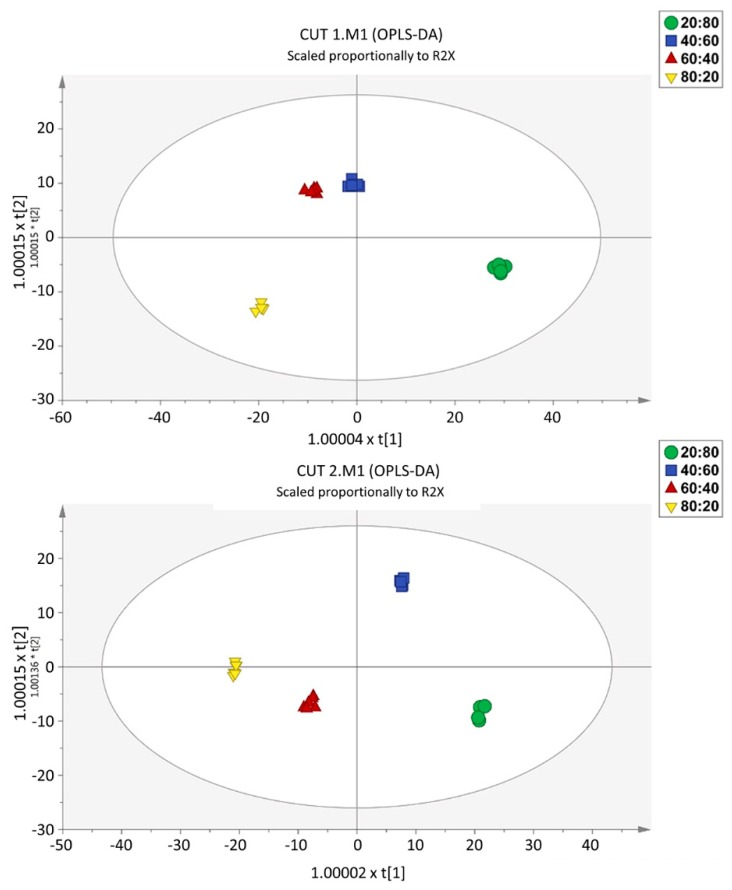
Score plots of Orthogonal Projection to Latent Structures Discriminant Analysis (OPLS-DA) supervised modelling, carried out from untargeted metabolomic profiles of the CT1 (CUT 1, upper) and CT2 (CUT 2, lower).

To provide molecular insights into the separations of the different nutrient ratios within each CT, we used a supervised OPLS-DA multivariate approach. Taking into account the main effect of the CT factor, two distinct models were built, one per cutting time. The score plots illustrate that samples were well separated, implying a relevant effect of the different nutrient solutions on the metabolomic profiles (Figure 4). The model parameters are characterized by high values for goodness-of-fit (*R*^2^*Y* > 0.99) and goodness-of-prediction (*Q*^2^*Y* > 0.95), with CV-ANOVA cross validation *p* values of 2.05 × 10^−12^ and 1.76 × 10^−14^ for CT1 and CT2, respectively. Moreover, the multivariate analysis indicated that the most dissimilar sample was the 20:80 R and, considering that the horizontal direction of the scatter plot captures the highest between-groups variation, this feature is more evident for CT1. The data also indicated that the largest difference between the two cuts refers to the 80:20 and 60:40 ratios. At CT2, the difference between these two treatments was less pronounced.

The variables importance in projection (VIP) approach allowed us to identify the compounds possessing the highest discrimination potential in the OPLS predictive models. The VIP score was calculated as a weighted sum of the squared correlations between the OPLS-DA components and the original variables. Compounds were identified as discriminant when presenting a VIP score > 1 (Supplementary File S1). Overall, secondary metabolism was largely represented in both CT1 and CT2. However, some differences were found between cuts regarding the specific metabolites, such as, for instance, the hormones profile. CT1 showed auxins-related compounds as discriminants, while CT2 showed gibberellins as principal discriminants hormone class. On the other hand, phenylpropanoids were extensively represented in both CTs.

To focus on the difference in the metabolic profiles according to the NO_3_^−^:Cl^−^ ratios, significant compounds (Volcano Plot, *p* < 0.05 and |fold-change| > 2) were identified and listed in the Supplementary Table S3 for CT1 (*n* = 198) and the Supplementary Table S4 for CT2 (*n* = 175). Interpretations were done using the omic viewer tool of PlantCyc (Supplementary Figures S2 and S3).

Taking the 80:20 ratio as reference condition, the basil response differed between the NO_3_^−^:Cl^−^ treatments. The largest impact was found in plants treated with 20:80 NO_3_^−^:Cl^−^ solution, and variation in fold change well associated to a progressive response to the different NS for both cuts (Supplementary Figure S4). Among the significant metabolites, secondary metabolism appeared to play an important role in the response to NO_3_^−^:Cl^−^ ratios. Specifically, a large number of compounds belonging to isoprenoids, nitrogen-containing secondary metabolites and phenylpropanoids were differentially accumulated in both CT1 and CT2. The amount of some amino acids (aas) strongly declined with the reduction of nitrogen supply, such as the γ-l-glutamyl-l-cysteine, precursor of glutathione, and the NO precursors l-arginine and l-citrulline. A difference between the CTs was that at CT1, all the detected compound classified as “amino acids and derivatives” showed a |FC| > 2 (in relation to the higher nitrogen limitation, the 20:80 R), while at CT2, the quantitative effect was much more limited. The difference between the cuts is, for instance, evident considering l-arginine and its nitric oxide-cycle by-product, l-citrulline. Both were significantly reduced with decreasing NO_3_^−^:Cl^−^ ratios, however, at CT2 the effect is almost halved compared to CT1. The alteration on phenylpropanoids was complex, although an overall increase could be observed with decreasing NO_3_^−^:Cl^−^ ratios. While the majority of the detected discriminant compounds showed a large increase at 20:80 R in both CTs, other metabolites were reduced. This trend is especially evident for leaves harvested at CT2. The isoprenoid hormones that have been found to be reduced compared to the 80:20 R are in all cases gibberellins (with larger decreases at CT2). Similarly, other isoprenoids were repressed in CT1, although the phytoalexins monodeglucosyl Des-Acyl Avenacin A and lubimin were strongly elicitated in all the nutrient ratios. A strong difference between CT1 and CT2 was relative to the metabolites classified as “Lipids”. Compared to the 80:20 R, these compounds were up-accumulated in CT1 in all R while at CT2, lipids were both up- and down-regulated. However, the effect of the NO_3_^−^:Cl^−^ ratios was similar for both CTs, with limited differences between treatments and with a higher magnitude of variation for the 20:80R. Among vitamins, l-Ascorbate was strongly up-accumulated when the R was different from 80:20 and this compound did not display a significant relative variation according to the NS. Rosmarinate, a dominant hydroxycinnamic acid ester of the Nepetoideae (the subfamily of the Lamiaceae that includes basil), was another compound in higher concentration than the reference treatment. The increasing accumulation of this metabolite was consistent with decreasing NO_3_^−^:Cl^−^ ratios, but it showed a much higher relative response in CT2 (approx. 10-fold). Notably, a wide reprogramming of hormonal profile could be observed at R differing from 80:20, with some differences between CTs. In addition to the above-reported gibberellins, both abscisic acid and jasmonates decreased in the different NS compared to 80:20 R, whereas salicylate and methyl salicylate increased (especially at 80:20 R). However, brassinosteroids increased when the R was different from 80:20, but only at CT1.

## 3. Discussion

The composition of the nutrient solution in soilless cultivation is arguably the most important factor the affects yield and quality, with direct and visible effects, especially for leafy vegetables. While recent studies illustrated the alterations on basil leaves that depends on nutrient availability [17,18,19], little is known on the metabolomics’ modifications that underline these phenomena.

The impact on biomass production of increasing chlorine/salinity or nitrate fertilization has been previously established for numerous greenhouse vegetables, and those sensitive or moderately sensitive to salinity [20]. In this work, using a full factorial design, we evaluated the possible antagonistic anion-anion effects on the industrial yield (i.e., leaf and stem fresh weight) and on the leaf metabolome in basil. The two factors under investigation (R and CT) significantly affected the parameters under investigation. There was a statistically significant interaction between the effects of NS ratios and the cut on the accumulation of chlorine in leaves. Moreover, metabolomics indicated that the different NO_3_^−^:Cl^−^ ratios induce wide alterations in basil, and that this effect has dissimilarities considering the two harvests.

The relation between nitrate and chlorine is complex for plant biology. The presence of an antagonistic ion-ion uptake in many species is generally accepted, usually also explained considering possible nutrient deficiency induced by excessive Cl^−^ [21,22]. High levels of chloride in the root zone may compete with NO_3_^−^ for the same channels and decrease the root-to-shoot translocation of NO_3_^−^ [23]. Salinity stress can stunt plant growth by lowering the demand for nitrogen and thereby downregulating the rate of nitrate uptake [24]. Moreover, it has been shown that the interplay between nitrate and chlorine is dose-dependent. For instance, nitrate levels in shoots were significantly affected only by the high level of Cl^−^ in Brassica [25].

The analysis of the mineral content in leaves indicated that a considerable amount of nitrate was taken up from the NS, and it was accumulated in leaf cells consistent with its amount in the NS. This trend was significantly affected by the amount of chlorine. A reduction of the nitrate uptake in leaves was previously reported, also for other leafy greens [26,27]. In this work, while the chorine content in leaves well correlated with its increasing amount in the NS, a large nitrate reduction was present at the highest chlorine concentration. We employed chlorine concentrations that were not expected to induce visible toxicity symptoms (e.g., chlorosis, leaf tip burning, stunting, etc.) [28]. In addition to an anion-anion competition, the linear decrease of the nitrate concentration from highest to intermediate NS ratios is likely to be due also to the chloride interference with several steps of nitrogen utilization (e.g., uptake, assimilation, translocation and remobilization). At the highest chlorine concentration, the marked effect on nitrate probably comes from the oxidative stress of the plant [20,29]. The mineral content also showed a significant variation for the CT factor only for the chlorine, which almost doubled in the stem and leaves that grew after the first cut. Conversely, nitrate accumulation in leaves at the second cut was not significantly different, or in relation to different NSs. The overall reduced, yet significant, decrease in yield at the second cut cannot be attributed to nitrate availability, being the accumulation of chlorine in leaves the only variable factor.

To verify the effects of our treatments in leaves at molecular level, we identified and analyzed the expression level of two putative nitrate transporters (NT). The scarcity of functionally characterized *O. basilicum* genes prompted a bioinformatics analysis as a starting point. For instance, the thaumatin-like protein ObTLP1 has been characterized on the basis of a similarity-based EST scan [30]. The number of retrieved sweet basil sequences, their length distribution and average size imply that the database we built is adequate to reliably annotate transcripts [31,32]. We identified 16 putative nitrate transporters of the NTR1 (NPF) family. This number should not be considered exhaustive, for two reasons. Firstly, there are usually 50 and more nitrate transporters in the currently sequenced dicotyledon genomes [33]. Moreover, the EST basil collections originate mainly from leaves (and in general, from above ground tissue such as stems and flowers) [34], and several transporters are mainly expressed in roots [35]. According to a phylogenetic analysis, five ESTs were classified as members of the NPF5 subfamily. This subfamily is the largest in plants, usually representing almost a quarter of the NTs [16]. Unfortunately, the high number of NTs in plants is arguably the main factor that hinders functional predictions within subfamilies. For instance, there is no clear correlation between a subfamily, a selectivity towards a substrate, or a biological function [16,36]. Transporters that share a high level of sequence similarity may exhibit different patterns of tissue expression, suggesting that they perform different functions [37].

The expression analysis of the two putative nitrate transporters (ObNPF1.1 and ObNPF5.2) confirmed their expression in leaves and revealed differences according to the NS ratios. A clear distinction was present for the extreme R. The transcriptional regulation of nitrate transporters is complex. A plethora of factors can induce the transcription of NTs, although much information is linked to lateral root growth and N-uptake and starvation [38,39]. The observed upregulation by nitrate in leaves is consistent with a possible role in NO_3_^−^ transport or removal from the xylem [40]. Nonetheless, the expression of NTs is also affected by stress and in particular, by chlorine ions. The concomitant variation in nitrate and chlorine concentration in leaves may account for the limited relative differences among conditions.

Considering also the limited pre-existing knowledge, we used an untargeted approach to have a broader view of the biochemical pathways that may be affected. The number of metabolites, their parent chemical class and their quantitative variation suggest that the metabolite composition of basil leaves is largely affected by the cultivation condition. Moreover, the progressive response to the different ratios at each cut indicate that the NS composition is an effective strategy to quantitatively modify the leaf metabolome in basil.

Among others, metabolic changes associated with processes involved in amino acids, lipids, secondary metabolites and hormones metabolisms. As expected, nitrate and chloride levels in the nutrient solution did not only affect pathways that lead to synthesis of related compounds (e.g., amino acids, amides and organic acids, for nitrate; stress-dependent oxidative signals and compounds, for chlorine), but also pathways that include a wide number of different classes of metabolites. Furthermore, the number of differential metabolites and extent of regulation were more pronounced at CT1 than CT2. In the hydroponically grown herb sorrel (*Rumex acetosa* L.), plant material from the second cut had a reduction of secondary metabolites, likely because the plant is investing more resources for primary metabolism (i.e., growth) [41].

Irrespective of the effect of CTs, amino acids and derivatives, compounds were down accumulated at decreasing NO_3_^−^:Cl^−^ ratios. The metabolomic signatures appear to be beyond the availability of nitrate as a source of organic nitrogen. For instance, considering the single amino acids, a linear correlation was rarely evident, implying that their reduction is not only due to the limited nitrogen availability. Low nitrogen levels are expected to favor the accumulation of secondary metabolites such as phenylpropanoids [42,43]. In our dataset of discriminant compounds, as expected, an overall increase of phenylpropanoid and other secondary metabolites was observed, but this phenomenon was more marked at the first cut. Moreover, some phenylpropanoids decreased with lowering NO_3_^−^:Cl^−^ ratios [44,45]. A complex alteration was also observed for the lipids’ profile, with sterols and phospholipids changing their accumulation from CT1 to CT2.

Cues of the progressive occurrence of an oxidative stress with decreasing NO_3_^−^:Cl^−^ ratios could be gained from differential compounds. These indications include the increase of radical scavengers (namely, phenylpropanoids, ascorbate/dehydroascorbate ratio, glutathione intermediates and tocotrienols) and the concurrent alteration of epoxy-PUFA and dihydroxycarotenes, which are mainly associated to an altered cellular redox state. Moreover, the decreased arginine and citrulline content suggests an impaired NO production. This is also supported by the decrease in polyamine (triferuloyl spermidine) at increasing chloride levels [46]. Although the impact of the cross-talk between ROS and NO is not well understood for leaf growth and development [47], it is likely that their interaction synergistically promotes cellular response to stress [48]. The observed concurrent increase in salicylic acid, an upstream regulator of both NO and flavonoids biosynthesis, is also coherent with previous findings [49]. In basil, different stresses increase phenolic compounds [50,51,52], with caffeic acid and its derivative ester (rosmarinic acid) being highly variable in those conditions [18,53]. In our dataset, the amount of rosmarinate largely associated with the chlorine concentration in leaves, being present in higher quantity at the second cut and consistently decreasing with increasing NO_3_^−^:Cl^−^ ratios. Overall, the metabolomics of the stress-related compounds, as well as the distinct position of the 20:80 R metabolomics’ profiles at both cuts, support the idea that the observed changes are also due to an adaptive response to nutrient-induced stress and not only to variation in the nutrient availability.

Finally, the presence of a wide metabolic reprogramming in basil is also supported by the changes in phyto-hormones. In addition to salicylate, jasmonates, brassinosteroids, gibberellins and abscisic acid were involved in the response to the factors under investigation. It is well known that the interplay between these signal molecules involves a number of plant functions, and therefore, the crosstalk between the nitrate, NO and phytohormones signaling pathways chains the multifaceted and complex metabolomic signatures we observed [46,54,55,56]

In conclusion, our work indicated that the interplay between nitrates and chlorines exerts a large effect on basil yield and metabolomic profile, that cannot be satisfactorily explained only by an anion-anion antagonist outcome (e.g., a replacement effects and carrier competition processes). Considerable changes were observed in a number of chemical classes and their relation with the different nutrient ratios suggests a dose-dependent effect that is mainly the combination of a response to nutrient availability (e.g., primarily nitrogen) [56] and an inducible response to stress, which is evident when chloride concentration in the nutrient solution exceeds that which is capable to satisfy nutrient requirements [28]. Our work highlighted the potential and the magnitude of the effect of NS management for leafy vegetables. The diversity of biochemical reactions captured in our study represent not only a contribution towards understanding the metabolic response to anion antagonism in plants, but also a resource for the identification and modulation of key factors and tools that will ultimately improve basil yield and quality.

## 4. Materials and Methods

### 4.1. Plant Material and Experimental Treatments

The experiment was carried out in the spring-summer growing season of 2016 in a glasshouse at the Experimental Station of the Department of Agricultural Sciences, University of Naples Federico II, located in Bellizzi (SA), Southern Italy. The sweet basil (*Ocimum basilicum* L.) seedlings were transplanted on April 28th, at the three true leaf stage, in pots containing 1.3 L of peat:perlite mixture in a 2:1 v/v ratio. Plastic pots were disposed in single rows at a plant density of 23 plants per square meter. Inside the glasshouse, the mean air temperature amounted to 26 °C, ranging from 16 to 33 °C. The relative humidity was 57 % (resp. 80%) during day (resp. night).

A factorial combination of different NO_3_^−^:Cl^−^ ratio (R) in the nutrient solution, and harvests (CT) was adopted. Factor R had four levels, namely 80:20, 60:40, 40:60 and 20:80 NO_3_^−^:Cl^−^. and factor CT two (CT1 and CT2). The experimental design was a randomized complete-block design with three replicates, yielding 24 experimental units (4 R × 2 CT × 3 replicates). Plastic pots were placed on an aluminum bench and each experimental unit (plot) accommodated 10 plants (150 total plants), of which the first two and the last two were considered guard plants and discarded after harvest. The nutrient solution was a modified Hoagland formulation, with the following composition: 1.5 mM phosphorus, 4.5 mM potassium, 6.5 mM calcium, 2.0 mM magnesium, 20 μM iron, 9 μM manganese, 0.3 μM cupper, 1.6 μM zinc, 20 μM boron, and 0.3 μM molybdenum, with an electrical conductivity (EC) and pH of 2.0 ± 0.1 dS m^−1^ and 6.0 ± 0.2, respectively. The four NO_3_^−^:Cl^−^ ratios were obtained by adding different amounts of Ca(NO_3_)_2_ and CaCl_2_. Specifically, nitrate concentration (NO_3_^−^; mM) and salt quantity (Ca(NO_3_)_2_ and CaCl_2_; mg L^−1^) of the four treatments in the nutrient solution were:

NO_3_^−^:Cl^−^ (80:20): 9.6 mM, 867.1 and 133.2 mg L^−1^, respectively

NO_3_^−^:Cl^−^ (60:40): 7.2 mM, 650.3 and 266.4 mg L^−1^, respectively

NO_3_^−^:Cl^−^ (40:60): 4.8 mM, 433.5 and 399.6 mg L^−1^, respectively

NO_3_^−^:Cl^−^ (20:80): 2.4 mM, 216.8 and 532.8 mg L^−1^, respectively

### 4.2. Biomass Determination, Nitrate and Chloride Analysis

At 47 (CT1) and 75 days after transplanting (CT2), six plants per experimental plot were harvested and leaves were separated from stems. At each harvest, the fresh shoot biomass (leaves + stems) were recorded. Leaf samples were dried in a forced-air oven at 70 °C for 72 h until constant weight and conserved for mineral analysis. The dried leaf tissue of the first and second harvest was used for nitrate and chloride analysis. The basil leaf tissue was pulverized using a cutting-grinded head (IKA, MF10.1, Staufen, Germany). The powder was extracted in Milli-Q water (Merck Millipore, Darmstadt, Germany) for 10 min at 80 °C in a thermostatic bath (ShakeTemp SW22, Julabo, Germany) and centrifuged at 6000 rpm for 10 min. The samples were filtered (0.20 µm) and stored at −20 °C as reported [57]. A Dionex ICS-3000 system (Sunnyvale, CA, USA) equipped with suppressed conductivity detection was used to determine the nitrate and chloride content of the samples. The two analyzed anions were separated on an IonPac AS11-HC column (250 × 4 mm), with a potassium hydroxide gradient eluent (flow rate 1.5 mL min^−1^). The two anions were expressed in g per kg dry weight.

### 4.3. In Silico Identification of Nitrate Transporters in O. basilicum

The database of proteins associated to the terms “nitrate transporter” (*n* = 2330) and “NRT1” (*n* = 8713) in Viridiplante (Taxonomy ID: 33090) NCBI was built from NCBI. Duplicated entries were removed considering the “Identifier”, yielding 9270 protein sequences. The database of *O. basilicum* transcripts comprises 23.845 ESTs and 85 cDNAs, also retrieved from NCBI. For the classification of the putative nitrate transporters, we performed a phylogenetic analysis. We retrieved protein sequences classified as NTR1 in Arabidopsis (*n* = 101) from the NCBI “Identical Protein Groups” resource, which include annotated coding regions from GenBank and RefSeq, and entries from SwissProt and PDB. Duplicates (e.g., isoforms, synonyms and homonyms) were removed through Entrez Batch. Proteins noted as “unknown”, “similar”, “Major facilitator superfamily protein”, “hypothetical” and “peptide transporter” were also excluded. The remaining 45 Arabidopsis proteins were subjected to multiple alignment using MUSCLE [58], along with the longest ORFs the basil ESTs that fulfilled the following criteria: classified as nitrate transporter sensu stricto (see Results); and e-value < 1 × 10^−50^. The phylogenetic reconstruction was conducted with the Maximum likelihood algorithm using the Jones–Taylor–Thorton amino acid substitution model (JTT) and assuming a substitution rate between the identical sites. Given the high similarity among the sequences and the fact that ORFs does not necessarily code for a full-length protein, the “Gaps/Missing Data” treatment was performed with the “partial deletion” option, using MEGA 7.0 software [59]. Inference of the phylogenetic tree was carried out with the nearest-neighbor-interchange (NNI) heuristic method. The basil transporters were named as ObNPFX.Y, where X indicates the subfamily and Y the single member within the species, as reported [16].

### 4.4. Gene Expression Analysis

Plant tissues were immediately frozen in liquid nitrogen and stored at −80 °C. RNA isolation, DNAse I treatment and first-strand cDNA synthesis were performed as described [60]. Real-time PCR was carried out in a final volume of 12.5 microl, containing the 6.5 μL of 2× QuantiFast SYBR Green PCR Master Mix (Qiagen), the primer pairs (300 nM) and the cDNA template. Amplifications were performed as reported [61]. Primers and their temperature of annealing are presented in Supplementary Table S1. Real-time RT-PCR was performed in three biological replicates. Reactions were carried out in three technical replicates in an ABI 7900 HT thermocycler (Applied Biosystems, Foster City, CA, USA). For quantification of gene expression, we employed the DeltaDeltaCt method [62], using the GAPDH as reference gene and the plants treated with the 20:80 ratio as calibrator genotype. Fold change are expressed in relative quantities (RQ) compared to the calibrator condition, set as 1. Significant differences were assessed with an analysis of variance, followed by Duncan’s post-hoc test for multiple comparisons of the 2^−ΔCt^ values.

### 4.5. Untargeted Metabolomics

Samples (1.0 g) were extracted in 10 mL of 0.1% formic acid in 80% aqueous methanol using an Ultra-Turrax (Ika T-25, Staufen, Germany) and centrifuged (12,000× *g*). The metabolic profile was investigated by using UHPLC-QTOF mass spectrometry as previously reported [63]. Briefly, a JetStream electrospray ionization source and a G6550 QTOF coupled to a 1290 ultra-high-performance liquid chromatograph (Agilent technologies, Santa Clara, CA, USA) were used. Reverse phase chromatography was carried out on an Agilent Zorbax Eclipse-plus C18 column (100 × 2.1 mm, 1.8 μm) using linear elution using acetonitrile in water (6% to 94%) as a mobile phase, both acidified with formic acid, in 33 min with flow rate 200 μL min^−1^). The mass spectrometer was operated in SCAN mode (100–1000 *m*/*z*) and positive polarity.

Raw spectral data were processes by means of Agilent Profinder B.06 software, using the targeted ‘find-by-formula’ algorithm, followed by mass (5 ppm) and retention time (0.05 min) alignment [64]. Compounds were putatively annotated by the combination of monoisotopic mass and isotopes ratio and spacing, according to Level 2 with reference to COSMOS Metabolomics Standards Initiative [65].

The compounds annotated in at least 75% of replications within at least one treatment were retained and used for post-acquisition. The reference database was PlantCyc 12.6 (Plant Metabolic Network, http://www.plantcyc.org; downloaded April 2018).

### 4.6. Statistical Analysis

Statistical analysis was carried out with the SPSS Statistics 20 software (IBM, Armonk, NY, USA). The plant biomass and mineral composition analysis were subjected to a two-way ANOVA. The mean values were separated according to Duncan’s test with *p* < 0.05. Variables relative to the two cuts were compared by a Student’s *t*-test. For the gene expression, statistical differences were assessed by an ANOVA, followed by Duncan’s post-hoc test for multiple comparisons, of the 2^ΔCt^ values. For the metabolomics analysis, Mass Profiler Professional B.12.06 was used for the chemometric interpretation of metabolomics dataset as previously described [66]. Compounds abundance was Log2 transformed, normalized at the 75th percentile, and baselined against the median. Differential compounds were investigated through Volcano plot analysis, combining fold-change (FC > 2) and ANOVA (*p* < 0.05, Bonferroni multiple testing correction). Differential compounds were then interpreted using the omic viewer pathway tool of PlantCyc, to identify pathways and processes from the metabolite list [67]. Venn analysis was carried out by using the software Venny 2.1 (https://bioinfogp.cnb.csic.es/tools/venny/). An unsupervised hierarchical cluster analysis was based on fold-change values and the similarity was set as ‘Euclidean’ and the ‘Wards’ linkage rule was chosen. Orthogonal Projections to Latent Structures Discriminant Analysis (OPLS-DA) supervised analysis was carried out loading the dataset into SIMCA 13 (Umetrics, Sweden). CV-ANOVA (*p* < 0.01) and permutation testing (*n* = 100) were also applied to validate and to exclude overfitting and outliers were investigated using Hotelling’s T2 (95% and 99% confidence limits for suspect and strong outliers, respectively) Goodness-of-fit R2Y and goodness-of-prediction Q2Y were also calculated from the OPLS-DA model. Subsequently, a variable importance in projection (VIP) analysis was used to select the most discriminant compounds.

## Figures and Tables

**Table 1:** Influence of NO_3_^−^: Cl^−^ ratios and cuts on basil yield and leaf mineral composition (mean ± standard error of the mean).

Source of Variance	Fresh Shoot Biomass	NO_3_^−^	Cl^−^
	(g shoot^−1^)	(g kg^−1^ dw)	(g kg^−1^ dw)
NO_3_^−^: Cl^−^ ratio (R)			
80:20	113.2 ± 4.43 a	32.13 ± 4.32 a	5.00 ± 0.89 d
60:40	108.7 ± 6.15 a	28.66 ± 1.53 a	11.66 ± 1.51 c
40:60	96.1 ± 4.95 b	15.95 ± 1.22 b	21.55 ± 4.41 b
20:80	70.1 ± 1.90 c	1.08 ± 0.23 c	40.89 ± 6.16 a
	***	***	***
Cuts (CT)			
CT1	105.2 ± 6.14	19.46 ± 4.20	13.01 ± 2.78
CT2	88.8 ± 4.53	19.45 ± 3.81	26.54 ± 5.57
t-value	*	ns	*
R × CT			
80:20 × CT1	121.9 ± 0.78	32.09 ± 8.76	3.90 ± 1.61 e
60:40 × CT1	119.7 ± 7.21	28.96 ± 2.77	8.37 ± 0.72 de
40:60 × CT1	106.0 ± 4.27	15.62 ± 2.01	12.44 ± 2.76 cd
20:80 × CT1	73.2 ± 1.58	1.15 ± 0.46	27.34 ± 2.05 b
80:20 × CT2	104.4 ± 4.58	32.17 ± 4.07	6.11 ± 0.39 e
60:40 × CT2	97.7 ± 3.96	28.35 ± 1.97	14.95 ± 0.34 c
40:60 × CT2	86.1 ± 2.43	16.28 ± 1.82	30.66 ± 2.56 b
20:80 × CT2	67.0 ± 2.46	1.01 ± 0.23	54.44 ± 1.25 a
	ns	ns	***

Legend: ns, *, ***: non-significant, significant at *p* ≤ 0.05, and *p* ≤ 0.001, respectively. Different letters (a, b, c, d and e) within each column indicate significant differences according to Duncan’s test (α = 0.05). The significance between the two cuts was evaluated with a Student’s *t*-test.

## Supplementary Materials

Supplementary materials can be found at https://www.mdpi.com/1422-0067/21/7/2482/s1. Figure S1: Histogram of the length distribution (nt) of the *O. basilicum* transcript database employed for the identification of putative nitrate transporters. Figure S2: Biosynthesis processes involved in plant response to treatments for CT1. The metabolomic dataset produced through UHPLC-ESI/QTOF-MS was subjected to Volcano Plot analysis (*p* < 0.05, fold-change > 2) and differential metabolites loaded into PlantCyc Pathway Tool (https://www.plantcyc.org/). The x-axis represents each set of subcategories while the y-axis corresponds to the cumulative fold-change. Figure S3: Biosynthesis processes involved in plant response to treatments for CT2. The metabolomic dataset produced through UHPLC-ESI/QTOF-MS was subjected to Volcano Plot analysis (*p* < 0.05, fold-change > 2) and differential metabolites loaded into PlantCyc Pathway Tool (https://www.plantcyc.org/). The x-axis represents each set of subcategories while the y-axis corresponds to the cumulative fold-change. Figure S4: Heatmap of the Fold Changes of the discriminant metabolites for the nutrient ratios (R) 60:40, 40:60 and 20:80 NO_3_^−^/Cl^−^ in comparison with the 80:20 NO_3_^−^/Cl^−^, for the two cuts (CT1 and CT2). Gradation from red to green is according to the colour scale at the bottom. For each cut, conditions are clustered (UPGMA) on the basis of Euclidean distances.

## Abbreviations

CT	cut
EST	Expressed Sequence Tag
HCA	Hierarchical Cluster Analysis
NCBI	National Center for Biotechnology Information
NS	Nutrient solution
NT	Nitrate Transporter
OPLS-DA	Orthogonal Projection to Latent Structures Discriminant Analysis
QTOF	Quadrupole Time-Of-Flight
R	NO_3_^−^: Cl^−^ ratio
ROS	Reactive Oxygen Species
UHPLC	Ultra High-Performance Liquid Chromatography
VIP	Variables Importance in Projection
