# Supplementary material for: Metabolic Insights into the Anion-Anion Antagonism in Sweet Basil: Effects of Different Nitrate/Chloride Ratios in the Nutrient Solution
Source: Int J Mol Sci. 2020 Apr 3;21(7):2482. doi: 10.3390/ijms21072482 (PMC7177776; doi:10.3390/ijms21072482)
Supplement: Supplementary file 1 [file ijms-21-02482-s001.zip › Supplementary Files/Supplementary Tables.docx]

Supplementary Table 1: Identification of *O.basilicum* unique hits classified as nitrate transporters.

The table shows for the 16 "best-blast-hits" classified as “nitrate transporters” the GenBank accession number of best-hit-protein, the number of basil ESTs that yielded a significant match, the representative Genbank accession of the *O. basilicum* EST, its length, number of blastx matched in the protein database, the description of the best balst hit, the e-value and similarity the GenBank accession of an EST of representative basil (ObEST), its length, the number of match that such EST has found in the date bank employed (the nitrate transporters in the Viridiplantae), the description of the best-blast-hit, the e-value and the similarity.

| Best Blast Hit (Genbank) | EST (n) | ObEST (GenBank) | Lenght (bp) | Number of blastx matches | Description | e-value | Similarity (%) |
| --- | --- | --- | --- | --- | --- | --- | --- |
| BAS86882 | 1 | DY336697 | 612 | 9 | Os03g0800000 [Oryza sativa Japonica Group] | 2.3115E-37 | 64 |
| EMS56066 | 1 | DY331863 | 758 | 1 | Nitrate transporter 1 | 1.08E-21 | 92 |
| EOY18514 | 2 | DY340349 | 374 | 1 | Nitrate excretion transporter 1, putative [Theobroma cacao] | 7.89E-19 | 97 |
| KHN43749 | 1 | DY331561 | 832 | 1 | Nitrate transporter 1 | 5.69E-15 | 70 |
| KZV40365 | 1 | DY342386 | 830 | 1 | nitrate transporter [Dorcoceras hygrometricum] | 8.88E-18 | 91 |
| PHU01356 | 4 | DY326693 | 664 | 1 | Protein NRT1/ PTR FAMILY 1 | 6.52E-15 | 67 |
| PHU28694 | 3 | JZ190573 | 650 | 1 | Protein NRT1/ PTR FAMILY 7 | 8.98E-26 | 63 |
| POE48602 | 2 | DY324430 | 524 | 1 | protein nrt1/ ptr family 5 | 3.14E-64 | 91 |
| XP_006650507 | 24 | DY322776 | 499 | 2 | PREDICTED: protein NRT1/ PTR FAMILY 8 | 6.66E-34 | 93 |
| XP_011096252 | 4 | JZ190717 | 525 | 20 | protein NRT1/ PTR FAMILY 5 | 1.41E-61 | 80.45 |
| XP_012832116 | 2 | DY321956 | 721 | 20 | PREDICTED: protein NRT1/ PTR FAMILY 5 | 2.81E-45 | 71.05 |
| XP_012852995 | 2 | DY327927 | 857 | 20 | PREDICTED: protein NRT1/ PTR FAMILY 2 | 1.17E-148 | 87.65 |
| XP_012854855 | 2 | DY336468 | 877 | 20 | PREDICTED: protein NRT1/ PTR FAMILY 5 | 9.12E-118 | 84.5 |
| XP_015631789 | 9 | DY342792 | 789 | 3 | PREDICTED: protein NRT1/ PTR FAMILY 8 | 3.16E-37 | 68.33 |
| XP_022725712 | 4 | DY328287 | 831 | 1 | protein NRT1/ PTR FAMILY 4 | 1.38E-13 | 82 |
| XP_022864112 | 2 | DY328527 | 826 | 20 | protein NRT1/ PTR FAMILY 2 | 3.48E-132 | 85.05 |

Supplementary Table 2: Naming of the identified *O. basilicum* ESTs according to Leran et al. 2014.

| Gene ID | Proposed name |
| --- | --- |
| DY328527 | ObNPF2.1 |
| DY327927 | ObNPF1.1 |
| DY321956 | ObNPF5.1 |
| JZ190717 | ObNPF5.2 |
| DY336468 | ObNPF5.3 |

**Supplementary Table 3.** Discriminant metabolites from Volcano Analysis (p-value<0.05; FC>2) at CT1. An asterisk (*) indicates metabolites marked as VIP in OPLS-DA (VIP Score > 1.15).

| **Parent Class** | **Class** | **Compound** | **Log FC 20:80 vs 80:20** | **Log FC 40:60 vs 80:20** | **Log FC 60:40 vs 80:20** |
| --- | --- | --- | --- | --- | --- |
| **Amino Acids And Derivatives** |  | L-Isowillardiine | -5.65 | -5.65 | -5.66 |
|  |  | &Gamma;-L-Glutamyl-(<I>S</I>)-Allyl-L-Cysteinyl-Glycine | 2.79 | -0.34 | -0.63 |
|  |  | &Gamma;-L-Glutamyl-L-Cysteine | -17.58 | -17.51 | 0.15 |
|  |  | <I>N</I>-Hydroxy-L-Dihomomethionine | 23.17 | 0.00 | 0.00 |
|  |  | Alliin | -12.69 | 1.15 | 1.11 |
|  |  | Cysteine | -16.93 | -11.46 | -16.57 |
|  |  | Homoisoalliin | 23.02 | 23.33 | 0.00 |
|  |  | L-Arginine | -3.77 | -2.97 | -0.37 |
|  |  | L-Citrulline | -21.89 | -22.05 | -21.68 |
|  |  | Phenylalanine | -2.83 | -2.44 | -0.67 |
| **Aromatic Compound** |  | 3,4-Dihydroxy-5-<I>All-Trans</I>-Hexaprenylbenzoate* | 17.65 | 12.20 | 13.38 |
|  |  | 3,4-Dihydroxy-5-<I>All-Trans</I>-Nonaprenylbenzoate | 17.51 | -0.20 | 0.23 |
|  |  | Demethylphylloquinol | 20.65 | 19.72 | 0.00 |
|  |  | Dihydrocoumarin* | -19.63 | -8.16 | 0.36 |
|  |  | Salicortin | -1.02 | -0.53 | 0.06 |
| **Carboxy Acids** |  | (9Z,13S,15Z)-12,13-Epoxyoctadeca-9,11,15-Trienoate/12-Oxo-Cis-10,15-Phytodienoate/Colnelenate/9,10-Epoxy-10,12Z,15Z-Octadecatrienoate | -18.28 | -0.49 | -0.18 |
|  |  | (9Z,15Z)-12,13-Epoxy-Octadeca-9,15-Dienoate | -1.20 | -1.61 | -0.25 |
|  |  | 2-Oxoadipate | 17.68 | -0.29 | 0.15 |
|  |  | 2-Oxoglutarate* | -17.04 | -11.54 | -16.67 |
|  |  | Acetonedicarboxylate* | -17.04 | -11.54 | -16.67 |
|  |  | Dimorphecolate/Vernolate/Densipolate/9,10-Epoxy-12-Cis-Octadecenoate/18-Oxo-Oleate | -1.51 | -1.23 | -0.56 |
|  |  | Propionyl Adenylate | 0.04 | 0.16 | 1.06 |
|  |  | Sterculate | -2.45 | -1.14 | -0.26 |
| **Hormones** | Brassinosteroids | Campestanol | 6.29 | -0.20 | 12.28 |
|  | Gibberellins | Gibberellin A<Sub>14</Sub>-Aldehyde | -9.12 | -9.45 | -0.02 |
|  |  | Gibberellin A<Sub>15</Sub> (Closed Lactone Form) | -18.32 | -12.68 | -0.02 |
|  |  | Gibberellin A<Sub>44</Sub> (Closed Lactone Form) | -3.44 | -2.28 | -1.02 |
| **Isoprenoids** | Carotenoids | &Beta;-D-Gentiobiosyl Crocetin | -1.10 | -0.29 | -0.27 |
|  |  | Bis(&Beta;-D-Glucosyl) Crocetin | -1.14 | -0.28 | -0.27 |
|  |  | Bixin Dimethyl Ester* | -20.91 | -1.32 | -1.30 |
|  |  | Capsanthin/Antheraxanthin/4-Hydroxyzeaxanthin | -0.05 | 3.38 | 0.15 |
|  |  | Lactucaxanthin/Zeaxanthin/Lutein/4,4-Dihydroxy-&Beta;,&Beta;-Carotene | 12.58 | 8.87 | -5.62 |
|  | Diterpenoids | <I>Syn</I>-Copalyl Diphosphate | -4.31 | -0.42 | 0.39 |
|  |  | 19-<I>O</I>-&Beta;-Glucopyranosyl-Steviol* | 9.92 | 10.85 | 10.22 |
|  |  | Steviolmonoside* | 19.87 | 13.71 | 6.73 |
|  | Sesquiterpenoids | Artemisinin* | -13.87 | -2.08 | -10.17 |
|  |  | Lubimin | 11.78 | 18.91 | 18.44 |
|  | Monoterpenol | (6<I>E</I>)-8-Hydroxygeraniol | 1.29 | 0.78 | 0.00 |
|  | Steroids | 4&Alpha;-Hydroxymethyl-4&Beta;-Methyl-5&Alpha;-Cholesta-8,24-Dien-3&Beta;-Ol/4&Alpha;-Hydroxymethyl-Ergosta-7,24(24<Sup>1</Sup>)-Dien-3&Beta;-Ol | -1.12 | -0.49 | 0.13 |
|  |  | Dehydrodesmosterol/Zymosterone/5&Alpha;-Cholesta-7,24-Dien-3-One | -19.42 | -19.18 | -18.97 |
|  | Terpenoid Derivative | (<I>E,E</I>)-Farnesylacetone | -18.79 | -12.89 | -12.60 |
|  | Tetraterpenoids | (2<I>E</I>,14<I>E</I>,18<I>E</I>)-Lycopatriene | -19.58 | -9.29 | -0.26 |
|  | Triterpenoids | Monodeglucosyl Des-Acyl Avenacin A* | 19.01 | 19.63 | 19.85 |
| **Lipids** |  | 1-[18-Hydroxyoeoyl]-2-[18-Hydroxy-Lioleoyl]-<I>Sn</I>-Glycerol | 18.28 | 14.61 | 6.20 |
|  |  | 1-18:1-2-16:1-Monogalactosyldiacylglycerol | 1.07 | 1.62 | 1.55 |
|  |  | 1-18:2-2-18:2-Monogalactosyldiacylglycerol* | 1.17 | 2.09 | 1.86 |
|  |  | 1-18:2-2-18:3-Digalactosyldiacylglycerol | 10.62 | 8.96 | 8.80 |
|  |  | 1-18:3-2-18:2-Digalactosyldiacylglycerol | 10.62 | 8.96 | 8.80 |
|  |  | Digalactosyldiacylglycerol | 17.25 | 0.15 | 6.04 |
|  |  | Dipalmitoyl Phosphatidate | -13.00 | 0.03 | 0.74 |
|  |  | Phosphatidylglycerophosphate (1-Octadecenoyl(9Z), 2-Palmitoyl) | 15.26 | 11.70 | 14.91 |
|  |  | Phosphatidylglycerophosphate (Dioctadec-9-Enoyl(Z)) * | -0.06 | 21.69 | 21.91 |
| **Nitrogen-Containing Secondary Metabolites** |  | &Gamma;-Chaconine/&Gamma;-Solanine | 13.92 | 0.12 | -6.23 |
|  |  | (S)-Tetrahydropapaverine/Laudanine | -3.41 | -7.62 | -1.68 |
|  |  | Betaxanthin | 12.18 | 11.88 | 0.89 |
|  |  | Chelirubine | -1.45 | 0.38 | 0.34 |
|  |  | Miraxanthin V* | -1.41 | -2.30 | -2.51 |
|  |  | Noscapine | 3.84 | 3.97 | 4.37 |
|  |  | Pumiloside | -19.05 | -1.43 | -0.63 |
|  |  | Swainsonine | -17.94 | -17.94 | 0.55 |
|  | Glucosinolates | 7-(Methylsulfinyl)Heptyl-Glucosinolate | -1.50 | -1.02 | -0.51 |
| **Phenylpropanoids** | Flavonoids | (-)-Epicatechin-3-<I>O</I>-Gallate | 0.07 | 0.31 | 1.20 |
|  |  | (-)-Phaseollin | 19.30 | 18.27 | 18.36 |
|  |  | (-)-Sativan | -2.82 | -1.74 | -1.67 |
|  |  | (2S)-Eriodictyol/2-Hydroxynaringenin/1-(4-Hydroxyphenyl)-3-(2,4,6-Trihydroxyphenyl)Propane-1,3-Dione/Dalbergioidin | 1.03 | -0.20 | 0.34 |
|  |  | 3,6,7,2',4'-Pentamethylquercetagetin/2'-Hydroxy 3,6,7,3',4'-Pentamethylquercetagetin | -1.02 | -0.54 | 0.11 |
|  |  | 7,4'-Dimethylquercetin | 19.04 | 0.00 | 0.00 |
|  |  | Dalcochinin-8'-O-&Beta;-Glucoside | -0.05 | -0.07 | 6.03 |
|  |  | Dalpatein | 17.70 | 17.85 | 6.11 |
|  |  | Isovitexin 2''-O-Rhamnoside/Vitexin 2''-<I>O</I>-&Beta;-L-Rhamnoside | 18.02 | 5.67 | 0.15 |
|  |  | Quercetagetin 7-<I>O</I>-Glucoside/Gossypetin 7-<I>O</I>-Glucoside | -1.54 | -1.03 | -0.51 |
|  |  | Scutellarin/Luteolin 7-O-&Beta;-D-Glucuronide | -1.55 | -1.01 | -0.47 |
|  |  | Syringetin/Eupatolitin/Dimethylmyricetin/Gossypetin 3',8-Dimethyl Ether/Quercetagetin 3',6-Dimethyl Ether | 13.37 | 12.47 | 12.26 |
|  | Lignans | (-)-4'-Demethyl-Deoxypodophyllotoxin | -1.54 | -0.44 | -0.35 |
|  |  | <I>O</I>4,<I>O</I>5--Dimethylthujaplicatin | -1.03 | -0.53 | 0.06 |
|  |  | Justicidin B/&Beta;-Maltose/Allolactose/Gibberellin A<Sub>8</Sub>-Catabolite/&Alpha;-Maltose/Fagopyritol/Melibiose/&Beta;-Gentiobiose/&Beta;-D-Cellobiose/Galactinol/&Alpha;-Lactose/Sucrose/&Alpha;,&Alpha;-Trehalose | -1.18 | -0.37 | -0.24 |
|  | Other Phenylpropanoids | 1-<I>O</I>-Vanilloyl-&Beta;-D-Glucose/Camptothecin | 1.29 | 0.47 | 0.28 |
|  |  | 4-Coumaraldehyde/1-Phenylpropane-1,2-Dione | -19.63 | -8.16 | 0.36 |
|  |  | Bergaptol/2-[(2'-Methylthio)Propyl]Maleate | -18.14 | -18.29 | -17.68 |
|  |  | Coumarinate/3-Hydroxy-<I>Trans</I>-Cinnamate/<I>Keto</I>-Phenylpyruvate/Enol-Phenylpyruvate/4-Coumarate/2-Coumarate | 2.31 | 1.13 | 1.15 |
|  | Other Phenylpropanoids | Daphnetin/Esculetin | 12.17 | -0.20 | 0.15 |
|  | Other Phenylpropanoids | Methylsalicylate/Azaguanine/Vanillin/<I>N</I>-Carbamoyl-L-Aspartate/3,4-Dihydroxyphenylacetaldehyde/Ferulate | 19.43 | 17.99 | 17.79 |
| **Secondary Metabolites** |  | Bisdemethoxycurcumin | 19.01 | -0.20 | 0.15 |
|  |  | Hyperxanthone E | 0.24 | 11.96 | 11.78 |
|  |  | Methoxyanigorufone | -2.79 | -1.71 | -1.64 |
| **Vitamins** |  | L-Ascorbate | 18.10 | 18.04 | 21.04 |
| **Others** |  | (3E)-Phytochromobilin/15,16-Dihydrobiliverdin/&Alpha;-Bilirubin/(3Z)-Phytochromobilin | 9.72 | -0.20 | 0.15 |
|  |  | &Alpha;-D-Glucuronate 1-Phosphate/&Alpha;-D-Galacturonate 1-Phosphate | 16.39 | 5.17 | 0.15 |
|  |  | &Gamma;-Glu-Cys-&Gamma;-Glu-Cys-&Beta;-Ala | 21.32 | -0.29 | -0.03 |
|  |  | &Gamma;-Thiomethyl Glutamate | 19.55 | 18.05 | 17.89 |
|  |  | (-)-Medicarpin-3-<I>O</I>-Glucoside | -0.64 | -1.43 | -0.04 |
|  |  | (+)-Pisatin/Robustaquinone B/1,7-Dihydroxy-6,8-Dimethoxy-2-Methylanthraquinone/<I>Trans</I>-Cinnamoyl-&Beta;-D-Glucoside/Dimethylkaempferol/2',7-Dihydroxy-4',5'-Dimethoxyisoflavone/Cirsimaritin/Ladanein | 1.98 | 1.24 | 1.26 |
|  |  | (<I>E</I>)-1-(L-Cysteinylglycin-<I>S</I>-Yl)-<I>N</I>-Hydroxy-&Omega;-(Methylsulfanyl)Hexan-1-Imine/1D-1-O-(Indol-3-Yl)Acetyl-Myo-Inositol/6-Hydroxy-Indole-3-Acetyl-Phenylalanine/Oxindole-3-Acetyl-Phenylalanine/Thenoyl Trifluoro Acetone/Hexanediol 1,6-Bisphosphate/4-Methyl-5-(2-Phosphooxyethyl)Thiazole | -0.22 | 17.12 | 0.15 |
|  |  | (<I>E</I>)-1-(L-Cysteinylglycin-<I>S</I>-Yl)-<I>N</I>-Hydroxy-2-(1<I>H</I>-Indol-3-Yl)Ethan-1-Imine | -1.30 | -0.38 | -0.27 |
|  |  | (<I>S</I>)-5-Hydroxyisourate | -18.26 | -18.42 | -12.12 |
|  |  | (2,6-Difluoro-4-Hydroxyphenyl)Pyruvate* | -1.56 | 0.19 | 0.59 |
|  |  | (2E)-2-Ethylidene-4-Hydroxy-5-Methyl-3(2H)-Furanone/3,5-Dihydroxyanisole | 2.30 | 1.09 | 1.08 |
|  |  | (2S)-2-Amino-4-Pentenoate/L-Proline | -1.66 | -1.05 | -0.50 |
|  |  | (Indol-3-Yl)Pyruvate* | -18.17 | -12.38 | -17.71 |
|  |  | (L-Cysteinylglycin-<I>S</I>-Yl)(1<I>H</I>-Indol-3-Yl)Acetonitrile | 2.19 | 1.45 | 1.47 |
|  |  | [(2S)-2-Amino-2-Carboxylatoethyl]-5-Hydroxy-2H-1,4-Benzothiazine-3-Carboxylate | 18.08 | 2.63 | 11.59 |
|  |  | <I>N,N',N''</I>-Triacetylchitotriose | -19.25 | -16.07 | -6.26 |
|  |  | <I>N</I><Sup>6</Sup>-(&Delta;<Sup>2</Sup>-Isopentenyl)-Adenosine 5'-Monophosphate | -0.16 | -0.32 | 5.77 |
|  |  | <I>N</I>-Vanillate-L-Glutamate | 8.21 | -0.38 | 0.15 |
|  |  | <I>N-P</I>-Tosyl-L-Phenylalanyl Chloromethyl Ketone | -13.00 | -0.59 | -0.41 |
|  |  | <I>S</I>-(Hydroxymethyl)Glutathione | 2.99 | 1.70 | 1.80 |
|  |  | <I>Sn</I>-1,2-Di(2-Propylpentanoyl)Glycerol | -18.49 | -14.21 | -18.25 |
|  |  | <I>Trans</I>-Coutarate/Phaselate | 18.35 | 8.47 | 11.91 |
|  |  | 1-(P-Butylphenyl)-2,2-Dimethyl-4,6-Diamino-1,2-Dihydro-S-Triazine* | -0.91 | -1.50 | 0.04 |
|  |  | 1,4-Dihydroxy-6,7,8-Trimethoxy-2-Methylanthraquinone/Robustaquinone H/Nevadensin/8-Hydroxy-Salvigenin/3,7,4'-Trimethylquercetin | 1.10 | 0.38 | 0.33 |
|  |  | 10-Deacetyl-2-Debenzoylbaccatin III | -18.61 | -18.32 | 0.93 |
|  |  | 1-16:0-2-18:3-Diacylglycerol-Trimethylhomoserine | 12.59 | 12.13 | 11.88 |
|  |  | 13-[(<I>E</I>)-2-Methylcrotonoyl]Oxylupanine* | -6.25 | -6.23 | 8.72 |
|  |  | 17-O-Deacetylvindoline | -0.18 | -0.03 | 6.69 |
|  |  | 1-Linoleoyl-2-&Alpha;-Linolenoyl-Phosphatidylcholine | 10.62 | 11.11 | 10.98 |
|  |  | 1-Phenyl-7-(3,4-Dihydroxyphenyl)-Hepta-1,3-Dien-5-One/(R)-Prunasin/Indican | -19.40 | -19.35 | -18.94 |
|  |  | 2-(2-Methylpyridin-3-Yl)Ethanol | -19.21 | -1.33 | 0.02 |
|  |  | 2-(8-Hydroxy-2-Oxotridecyl)-6-Oxopyran-4-Olate | -3.84 | -1.98 | -2.33 |
|  |  | 2,4-Dihydroxycinnamate | 2.22 | 1.03 | 1.11 |
|  |  | 2'-Deoxymugineate | -2.77 | -1.73 | -1.64 |
|  |  | 3-Dehydrosphinganine/Sphingosine | -1.75 | -1.69 | -1.24 |
|  |  | 4,5-<I>Seco</I>-Dopa* | -21.03 | 0.22 | 0.73 |
|  |  | 4-Amino-2,6-Dinitrotoluene | 19.54 | 18.66 | 14.71 |
|  |  | 4-Amino-2,6-Dinitrotoluene Glucoside* | -1.41 | 0.28 | 0.53 |
|  |  | 4-Nitrophenol | 1.76 | 0.42 | -0.20 |
|  |  | 4'-Phosphopantetheine Group | -11.36 | -11.34 | -5.12 |
|  |  | 4'-Phosphopantetheine/&Gamma;-L-Glutamyl-(<I>S</I>)-2-Carboxypropyl-L-Cysteine | -11.36 | -11.34 | -5.12 |
|  |  | 5 &Alpha;-Carboxystrictosidine | -1.35 | 0.17 | 0.28 |
|  |  | 5,7-Dihydroxy-2-Methylchromone/7-Hydroxy-8-Methoxycoumarin/Scopoletin/Juglone | 19.43 | 18.52 | 11.90 |
|  |  | 5-Mercuriocytidine | -1.73 | -1.04 | -0.43 |
|  |  | 5-Methyltetrahydrofolate Mono-L-Glutamate | -2.79 | -0.27 | -1.29 |
|  |  | 7,2'-Dihydroxy-4'-Methoxy-Isoflavanol Carbocation Intermediate 2* | -18.21 | -0.56 | -18.21 |
|  |  | 7-Deoxyloganin* | -1.22 | -1.62 | -0.47 |
|  |  | 9-Hydroxy-10-Oxo-12-Octadecenoate | -19.47 | -0.45 | -0.25 |
|  |  | Adenosine 5'-Phosphoramidate | 1.09 | 0.39 | 0.33 |
|  |  | Adenylyl-Imidodiphosphate | -1.21 | -0.20 | 0.25 |
|  |  | Adhyperforin/Echinenone | 19.16 | 12.57 | 0.12 |
|  |  | All-<I>Trans</I>-Hexaprenyl Diphosphate/Presqualene Diphosphate | -1.05 | 0.08 | 0.29 |
|  |  | Allosamidin | -2.98 | -0.28 | 0.03 |
|  |  | ATP/2-Hydroxy-Datp/Dgtp | -1.29 | -0.22 | 0.23 |
|  |  | Benzyl 6-<I>O</I>-&Beta;-<I>D</I>-Apiofuranosyl-&Beta;-<I>D</I>-Glucopyranoside/Benzyl Alcohol 6-<I>O</I>-&Beta;-<I>D</I>-Xylopyranosyl-&Beta;-<I>D</I>-Glucopyranoside | -1.03 | -0.53 | 0.06 |
|  |  | Berbamunine/Guattegaumerine | -2.50 | -0.39 | -0.04 |
|  |  | Bph-700 | -15.31 | 6.62 | 3.46 |
|  |  | Brassinosteroid/Triterpenoid/Soyasapogenol/Tocopherol | 19.70 | 18.73 | 0.00 |
|  |  | Coniferaldehyde Glucoside | 1.10 | 0.39 | 0.34 |
|  |  | D,L-&Alpha;-Methylphosphinothricin | -0.06 | -0.06 | 8.80 |
|  |  | D-Erythro-Imidazole-Glycerol-Phosphate | -1.79 | -0.11 | 0.17 |
|  |  | Diethyl Thiosulfinate | 2.25 | 0.44 | 0.30 |
|  |  | Dihydrochelirubine | 1.14 | 0.40 | 0.34 |
|  |  | Dihydrosanguinarine | 2.05 | 1.29 | 1.30 |
|  |  | Dimeric Urushiol | 6.19 | 7.11 | 3.59 |
|  |  | Dimethylallyl Diphosphate/Isopentenyl Diphosphate | 11.13 | 5.48 | -2.54 |
|  |  | Dump/2'-Deoxyuridine 3'-Monophosphate | 10.65 | -0.20 | 0.15 |
|  |  | Ehna | -1.48 | -1.50 | -0.98 |
|  |  | Epoxypheophorbide <I>A</I> | -1.05 | -0.10 | 0.30 |
|  |  | Esculin/N-Beta; -D-Glucosyl-Daphnetin/Daphnetin-8-Glucoside/N-Beta;-D-Glucosyl-Esculetin/Cichoriin/Daphnin/Esculin | -1.31 | 1.13 | 1.06 |
|  |  | Ethyleneglycol Phosphate/L-Threonate/L-Homocysteine/<I>S</I>-Methyl-L-Cysteine | 17.62 | 10.90 | 0.15 |
|  |  | Fluorofumarate | 18.90 | 18.02 | 0.21 |
|  |  | Fmnh<Sub>2</Sub> | -1.49 | -0.98 | -0.46 |
|  |  | HPOTE/(9Z,11E,14Z)-(13S)-Hydroperoxyoctadeca-(9,11,14)-Trienoate | -18.00 | 0.04 | 0.01 |
|  |  | Imidazole Acetol-Phosphate | -1.69 | -0.14 | 0.14 |
|  |  | Inosine/Abscisate/4-Prenylphlorisobutyrophenone/Dihydroxyphaseic Acid | -2.76 | -1.71 | -1.63 |
|  |  | Kinetin | -17.50 | -5.54 | -5.11 |
|  |  | L-Dehydro-Ascorbate | -0.44 | -0.55 | -3.45 |
|  |  | Linoleate Group | -1.70 | -1.65 | -1.21 |
|  |  | L-Phosphinothricin | 2.19 | 1.04 | 1.10 |
|  |  | L-Selenocystathionine | 16.46 | 5.22 | 0.15 |
|  |  | Lupiwighteone | -0.50 | 17.03 | 5.93 |
|  |  | Maltotriose/Raffinose/Kestotriose/Fagopyrito | -12.75 | -1.19 | -11.92 |
|  |  | Methylphoracetophenone/(R)-3-(4-Hydroxyphenyl)Lactate | 2.24 | 0.98 | 1.06 |
|  |  | Methylsuccinate/Glutarate | -19.14 | -4.24 | 0.26 |
|  |  | N-(Indol-3-Ylacetyl)-&Beta;-D-Glucose/Isowighteone | -0.31 | 17.03 | 0.05 |
|  |  | N<Sup>&Omega;</Sup>-Hydroxy-L-Arginine/3-Hydroxy-9-Apo-&Delta;-Caroten-9-One/3-Hydroxy-&Beta;-Ionone | -1.28 | -0.79 | -0.10 |
|  |  | Norbixin/(-)-Yatein/7-(Methylsulfanyl)Heptyl-Desulfoglucosinolate | -11.65 | -5.81 | -11.28 |
|  |  | Octyl &Beta;-D-Glucopyranoside | -20.21 | -7.21 | -0.11 |
|  |  | Pamidronate | 17.21 | -0.20 | 0.15 |
|  |  | P-Chlorophenylalanine* | -17.50 | -11.87 | -14.55 |
|  |  | Phenylacetaldehyde | -18.99 | -0.85 | 0.33 |
|  |  | Phenylthiourea | 19.40 | 0.07 | 4.70 |
|  |  | Pheophorbide <I>A</I> | -1.19 | 0.12 | 0.33 |
|  |  | Pheophorbide <I>B</I> | -19.88 | 0.10 | 0.35 |
|  |  | Phloretin/Apiforol/3-Isopropyl-10-(Methylthio)-2-Oxodecanoate/(+)-Afzelechin/Hemigossypolone/(3R,4R)-7,2',4'-Trihydroxyisoflavanol/(-)-Epiafzelechin | -15.64 | -12.76 | -9.50 |
|  |  | Pimelate | 2.17 | 1.08 | 1.07 |
|  |  | Pinocembrin Chalcone/Isoliquiritigenin/(2<I>S</I>)-Liquiritigenin/(6ar,11ar)-3,9-Dihydroxypterocarpan/(<I>S</I>)-Dihydrodaidzein/Emodin Anthrone | -15.44 | -15.40 | -14.99 |
|  |  | Prostaglandin | -4.03 | -1.63 | -0.40 |
|  |  | Protopine | -17.58 | -17.56 | -10.46 |
|  |  | Red Chlorophyll Catabolite | -16.64 | -11.88 | -3.50 |
|  |  | Rosmarinate | 3.08 | 1.74 | 1.83 |
|  |  | S-Adenosyl-1,8-Diamino-3-Thiooctane* | -11.72 | -11.71 | -11.36 |
|  |  | Salicylate/Hydroxybenzoate | 12.87 | -0.20 | 0.15 |
|  |  | Sanguinarine | 2.15 | 1.41 | 1.43 |
|  |  | S-Cheilanthifoline/(S)-Nandinine | 1.24 | 0.45 | 0.26 |
|  |  | Soyasaponin III* | 1.97 | 1.94 | 3.08 |
|  |  | Tellurite | -17.94 | -8.96 | -0.10 |
|  |  | Tetrahydropteroyl Mono-L-Glutamate* | -1.91 | -1.33 | 1.60 |
|  |  | Triferuloyl Spermidine* | -20.29 | -8.63 | -10.45 |
|  |  | Tubercidine 5'-Monophosphate | 1.46 | 1.71 | 1.16 |
|  |  | Umbelliferone/4-Hydroxycoumarin | 2.24 | 1.09 | 1.11 |

**Supplementary Table 4.** Discriminant metabolites from Volcano Analysis (p-value<0.05; FC>2) at CT2. An asterisk (*) indicates metabolites marked as VIP in OPLS-DA (VIP Score > 1.15).

| **Parent Class** | **Class** | **Compound** | **Log FC 20:80 vs 80:20** | **Log FC 40:60 vs 80:20** | **Log FC 60:40 vs 80:20** |
| --- | --- | --- | --- | --- | --- |
| **Amino Acids And Derivatives** |  | &Gamma;-L-Glutamyl-L-Cysteine | -18.10 | -18.06 | -18.06 |
|  |  | <I>N</I>-Acetyl-<I>S</I>-Geranylgeranyl-L-Cysteine | 1.54 | 1.00 | 0.04 |
|  |  | <I>N</I>-Hydroxytrihomomethionine | -1.64 | -0.95 | -0.29 |
|  |  | Buthionine Sulfoximine | -1.74 | -1.17 | -0.81 |
|  |  | L-Arginine | -2.03 | -0.69 | -0.41 |
|  |  | L-Citrulline | -10.28 | -10.77 | -10.24 |
|  |  | L-Homocysteine/L-Threonate/<I>S</I>-Methyl-L-Cysteine | 17.97 | 17.47 | 16.70 |
|  |  | L-Isowillardiine | -0.06 | 10.13 | -0.25 |
|  |  | L-Phenylalanine | -21.17 | -0.81 | -0.10 |
|  |  | Threonine/Serine | -12.32 | 5.70 | 5.82 |
| **Aromatic Compounds** |  | 4-Hydroxycoumarin | 2.68 | 1.38 | 0.66 |
|  |  | Amino-Dinitrotoluene | 18.23 | -0.19 | -0.21 |
|  |  | Coumaryl Acetate | -0.17 | 12.40 | 20.15 |
|  |  | DIMBOA | -10.97 | -0.10 | -1.02 |
|  |  | Dimeric Urushiol Peroxide | 18.07 | 5.86 | -0.21 |
|  |  | Indol-3-Ylmethylisothiocyanate-Glutathione | 0.94 | 1.13 | 0.44 |
| **Carboxy Acids** |  | &Alpha;,&Omega;-9<I>Z</I>-Octadecenedioate* | -19.13 | -9.94 | 0.56 |
|  |  | &Alpha;-Linolenate | -0.45 | -1.30 | 0.21 |
|  |  | &Gamma;-Linolenate/Punicate/Calendate/&Alpha;-Eleostearate/Crepenynate | -13.73 | -4.25 | -13.82 |
|  |  | (9Z,12Z)-Octadecadien-6-Ynoate | 15.73 | 6.32 | -0.21 |
|  |  | 2-[(2'-Methylthio)Propyl]Maleate | -17.56 | -17.62 | -17.48 |
|  |  | 2-Oxoadipate | 18.03 | 17.50 | 0.18 |
|  |  | Behenate | -0.03 | -0.19 | 17.94 |
|  |  | Linoleate | -0.66 | -1.04 | 0.21 |
|  |  | Phytanate/Arachidate | 1.02 | -0.17 | 0.66 |
|  |  | Sterculate | -2.80 | -2.54 | -1.08 |
| **Hormones** | Cytokinins | Dihydrozeatin-N-Glucoside | -1.01 | -0.56 | -1.30 |
|  | Gibberellins | Gibberellin A<Sub>15</Sub> (Closed Lactone Form) | -18.14 | -18.20 | -18.22 |
|  |  | Gibberellin A<Sub>24</Sub> | -1.85 | -1.51 | -0.43 |
|  |  | Gibberellin A<Sub>44</Sub> (Closed Lactone Form) | -2.11 | -1.55 | -0.71 |
|  |  | Methyl Gibberellin A/16&Alpha;, 17-Epoxy Gibberellin A | -20.38 | -20.38 | -0.83 |
| **Isoprenoids** | Carotenoids | &Beta;-Ionone | -2.89 | -1.09 | -0.77 |
|  |  | 3S,3'S-Astaxanthin | -15.83 | -3.33 | 3.14 |
|  |  | 4',4'-Dihydroxyadonixanthin/CHAPS | -15.98 | -3.48 | 2.79 |
|  |  | 4'-Hydroxyadonixanthin/3,4',4'-Trihydroxyechinenone | 2.23 | 1.28 | 1.06 |
|  |  | Bixin Aldehyde* | -14.88 | -14.95 | -14.97 |
|  |  | Bixin Dimethyl Ester | -19.64 | -19.57 | -0.74 |
|  |  | Crocetin | -1.85 | -1.51 | -0.42 |
|  | Diterpenoids | <I>Ent</I>-7&Alpha;-Hydroxykaur-16-En-19-Oate* | -16.88 | -13.55 | -20.34 |
|  |  | 19-<I>O</I>-&Beta;-Glucopyranosyl-Steviol | 10.64 | 10.50 | 0.00 |
|  |  | Oryzalide A | 1.75 | 1.10 | 0.80 |
|  |  | Steviolmonoside | 20.95 | 20.80 | -0.21 |
|  | Monoterpenes | Secologanin | -1.28 | 2.74 | 1.29 |
|  | Sesquiterpenoids | [4-(5,5-Dimethylcyclohex-1-En-1-Yl)-Cyclohex-1-En-1-Yl] Methanol | 3.57 | 3.50 | -17.89 |
|  |  | Artemisinin | -18.18 | -18.24 | -0.35 |
|  | Steroids | 4&Alpha;-Hydroxymethyl-4&Beta;-Methyl-5&Alpha;-Cholesta-8,24-Dien-3&Beta;-Ol/4&Alpha;-Hydroxymethyl-Ergosta-7,24(24<Sup>1</Sup>)-Dien-3&Beta;-Ol | -1.58 | -0.59 | -0.16 |
|  | Terpenoid Derivatives | (<I>E,E</I>)-Farnesylacetone | -11.73 | -11.89 | 6.42 |
|  |  | Hydroxytaxusin | -1.01 | 1.22 | 1.12 |
|  | Tetraterpenoids | (2<I>E</I>,14<I>E</I>,18<I>E</I>)-Lycopatriene* | -19.73 | -19.80 | -19.82 |
|  | Triterpenoids | Baccatin III | -12.44 | -12.24 | 6.31 |
|  |  | Glycyrrhetaldehyde | 2.39 | -6.23 | 11.38 |
|  |  | Presqualene Diphosphate | -1.69 | -1.29 | -0.57 |
| **Lipids** |  | 1-Linoleoyl-2-Linoleoyl-Phosphatidylcholine/1-&Alpha;-Linolenoyl-2-Oleoyl-Phosphatidylcholine/1-Oleoyl-2-&Alpha;-Linolenoyl-Phosphatidylcholine | -0.34 | 0.10 | -10.26 |
|  |  | 1-Oleoyl-2-Palmitoyl-Phosphatidylglycerol | 14.55 | -3.60 | -3.62 |
|  |  | 1-Palmitoyl-2-Linoleoyl-Phosphatidylcholine | -18.01 | -7.83 | -1.81 |
|  |  | 1-Palmitoylglycerol 3-Phosphate/1-Palmitoleylglycerol 3-Phosphate | -19.53 | -19.51 | -19.51 |
|  |  | Monogalactosyldiacylglycerol | -6.58 | -6.64 | 6.73 |
|  |  | Monogalactosyldiacylglycerol | 14.87 | -3.61 | -3.63 |
|  |  | Phosphatidylglycerophosphate (1-Octadecenoyl(9Z), 2-Palmitoyl) | 11.59 | 11.36 | 2.37 |
| **Nitrogen-Containing Secondary Metabolites** | Alkaloids | (S)-Tetrahydropapaverine/Laudanine | -18.58 | -19.38 | 0.24 |
|  |  | Betanidin | -6.84 | -7.66 | -19.89 |
|  |  | Dihydrochelerythrine | 1.27 | 1.03 | -1.03 |
| **Phenylpropanoids** | Flavonoids | (-)-Sativan | -1.91 | -1.58 | -0.27 |
|  |  | Afrormosin-7-O-Glucoside-6''-O-Malonate | 1.22 | 0.65 | -0.23 |
|  |  | Dalcochinin-8'-O-&Beta;-Glucoside | -18.16 | -9.75 | -6.09 |
|  |  | Dimethylquercetin/Pilosin | -20.51 | -20.79 | -0.33 |
|  |  | Gardenin B/Dalnigrein | -0.04 | 6.53 | -0.15 |
|  |  | Isovitexin 2''-O-Rhamnoside/Vitexin 2''-<I>O</I>-&Beta;-L-Rhamnoside | 18.16 | 11.91 | -0.21 |
|  |  | Patuletin/Dihydro-10-Thiopteroate/Laricitrin/3,3',4',5,7-Pentahydroxy-8-Methoxyflavone | -0.09 | 6.83 | -0.21 |
|  |  | Vitexin/Kaempferol-3-Rhamnoside/Pelargonidin-3-<I>O</I>-&Beta;-D-Glucoside/3-<I>C</I>-Glucosyl-2,4,4',6-Tetrahydroxydibenzoylmethane/6-<I>C</I>-Glucosyl-2-Hydroxynaringenin/Isovitexin | -1.07 | -1.44 | -0.51 |
|  | Lignans | (+)-Secoisolariciresinol Monoglucoside | -0.52 | 1.54 | 0.77 |
|  |  | Arctigenin* | -8.19 | -3.21 | 2.21 |
|  | Other Phenylpropanoids | Bergaptol | -17.55 | -17.62 | -17.48 |
|  |  | Coumarinate/3-Hydroxy-<I>Trans</I>-Cinnamate/<I>Keto</I>-Phenylpyruvate/Enol-Phenylpyruvate/Coumarate | 1.71 | 0.40 | -0.35 |
|  |  | Delphinidin/8-Hydroxykaempferol/Tricetin/Bracteatin/Quercetin/8-Hydroxy-Luteolin | 16.57 | 16.53 | 16.46 |
|  |  | Ferulate | 2.99 | 2.65 | 1.99 |
|  |  | Furcatin* | -6.12 | 6.06 | -6.20 |
|  |  | Methylsalicylate/Azaguanine/Vanillin/<I>N</I>-Carbamoyl-L-Aspartate/3,4-Dihydroxyphenylacetaldehyde | 3.11 | 2.76 | 2.11 |
|  |  | Umbelliferone | 13.03 | 11.73 | 11.01 |
| **Vitamin** |  | &Beta;-Tocotrienol | -9.21 | -4.01 | -9.52 |
|  |  | &Gamma;-Tocotrienol | -15.33 | -4.60 | -15.30 |
| **Others** |  | &Alpha;-3',4'-Anhydrovinblastine* | 0.86 | 1.55 | 0.55 |
|  |  | &Alpha;-D-Galacturonate 1-Phosphate | 16.77 | 16.60 | 13.41 |
|  |  | &Alpha;-D-Glucuronate 1-Phosphate | 16.77 | 16.60 | 13.41 |
|  |  | &Gamma;-Thiomethyl Glutamate | 3.11 | 2.76 | 2.09 |
|  |  | (-)-Medicarpin-3-<I>O</I>-Glucoside* | -0.27 | -2.06 | -0.33 |
|  |  | (<I>E</I>)-<I>N</I>-Hydroxy-5-(Methylsulfanyl)Pentimidothioate/Coniferaldehyde | -0.03 | 5.79 | 0.00 |
|  |  | (<I>E</I>)-1-(Glutathion-<I>S</I>-Yl)-<I>N</I>-Hydroxy-&Omega;-(Methylsulfanyl)Hexan-1-Imine | -0.56 | -1.09 | 0.00 |
|  |  | (<I>E</I>)-7-(Methyltsulfanyl)Heptanal Oxime | -21.87 | -1.91 | -21.99 |
|  |  | (<I>R</I>)-Pantolactone | -12.24 | -11.95 | -6.29 |
|  |  | (<I>S</I>)-5-Hydroxyisourate* | -17.51 | -17.57 | -17.43 |
|  |  | (<I>S</I>)-Scoulerine/(S)-Coreximine | -1.90 | -12.52 | -0.24 |
|  |  | (2,6-Difluoro-4-Hydroxyphenyl)Pyruvate | -2.48 | -0.77 | -0.62 |
|  |  | (2E)-2-Ethylidene-4-Hydroxy-5-Methyl-3(2H)-Furanone | 23.40 | 22.19 | 21.47 |
|  |  | (Indol-3-Yl)Pyruvate | -17.58 | -17.64 | -6.26 |
|  |  | (L-Cysteinylglycin-<I>S</I>-Yl)(1<I>H</I>-Indol-3-Yl)Acetonitrile | 2.97 | 1.64 | 0.00 |
|  |  | /(+)-Pisatin/1,7-Dihydroxy-6,8-Dimethoxy-2-Methylanthraquinone/Dimethylkaempferol/2',7-Dihydroxy-4',5'-Dimethoxyisoflavone/Cirsimaritin/Ladanein/ | 22.34 | 21.32 | 10.43 |
|  |  | /(3R,4R)-7,2',4'-Trihydroxyisoflavanolphloretin/Apiforol/3-Isopropyl-10-(Methylthio)-2-Oxodecanoate/(+)-Afzelechin/Hemigossypolone/(-)-Epiafzelechin | -11.81 | -11.88 | -6.04 |
|  |  | <I>N</I>-(2-Chloro-4-Pyridyl)-<I>N'</I>-Phenylurea | -1.46 | -1.56 | -1.22 |
|  |  | <I>N</I><Sup>6</Sup>-(&Delta;<Sup>2</Sup>-Isopentenyl)-Adenosine 5'-Monophosphate | 0.64 | 1.01 | 0.04 |
|  |  | <I>Sn</I>-1,2-Di(2-Propylpentanoyl)Glycerol | -18.05 | -12.37 | -9.57 |
|  |  | 1-(P-Butylphenyl)-2,2-Dimethyl-4,6-Diamino-1,2-Dihydro-S-Triazine | -2.15 | -0.28 | -1.77 |
|  |  | 1,2-Dehydroreticuline/Salutaridine/(S)-Corytuberine | -20.40 | -20.40 | -0.83 |
|  |  | 1,2-Dioctanoyl-1,2,6-Hexanetriol/Docosanedioate | -0.47 | 1.67 | -0.07 |
|  |  | 1,5-Dideoxy-1,5-Imino-D-Galactitol | -8.78 | -0.47 | -0.56 |
|  |  | 1-<I>O</I>-(4-Coumaroyl)-&Beta;-D-Glucose | 20.53 | 6.83 | -0.21 |
|  |  | 12-Hydroxydihydrochelirubine/(2<I>E</I>,6<I>E</I>)-Farnesyl Diphosphate | -0.19 | 12.99 | 13.02 |
|  |  | 1-Deoxymannojirimycin | -11.42 | -3.38 | -11.68 |
|  |  | 2-(2-Methylpyridin-3-Yl)Ethanol/Phenylacetaldehyde | -18.86 | -0.89 | -0.39 |
|  |  | 2-(8-Hydroxy-2-Oxotridecyl)-6-Oxopyran-4-Olate | -2.19 | -1.31 | -0.24 |
|  |  | 2,3-Dimethyl-6-Geranylgeranyl-1,4-Benzoquinol | -9.21 | -4.01 | -9.52 |
|  |  | 2,4-Dihydroxycinnamate | 3.66 | 2.37 | 1.69 |
|  |  | 2-Amino-6-Hydroxymethyl-7,7-Dimethyl-7,8-Dihydropteridin-4-One | -1.38 | -0.98 | -0.37 |
|  |  | 2-Benzyl-Thiohydroximate-O-Sulfate | -0.06 | 10.22 | -0.22 |
|  |  | 2'-Deoxymugineate | -1.86 | -1.55 | -0.27 |
|  |  | 2-Undecanone | -2.90 | -1.13 | -0.76 |
|  |  | 3,5-Dihydroxyanisole | 23.33 | 22.11 | 21.39 |
|  |  | 3-Chlorodiaminopimelate | -18.47 | -1.47 | -1.18 |
|  |  | 3-Isopropyl-6-(Methylthio)-2-Oxohexanoate* | -0.82 | -0.58 | -20.72 |
|  |  | 3-Methoxy-4-Hydroxy-5-<I>All-Trans</I>-Hexaprenylbenzoate | 2.16 | 1.22 | 0.99 |
|  |  | 4,5-<I>Seco</I>-Dopa | -20.99 | -13.88 | -21.08 |
|  |  | 4-Hydroxy-<I>Trans</I>-8-Sphingenine* | 1.07 | -0.17 | 0.70 |
|  |  | 4-Methyl-5-(2-Phosphooxyethyl)Thiazole* | -5.61 | 5.59 | 10.64 |
|  |  | 4-Nitrophenol | 2.18 | 0.99 | 0.35 |
|  |  | 5 &Alpha;-Carboxystrictosidine | -2.26 | -1.54 | -0.91 |
|  |  | 5,10-Methylenetetrahydropteroyl Tri-L-Glutamate* | -0.05 | 12.85 | -0.05 |
|  |  | 5,6-Epoxy-3-Hydroxy-9-Apo-&Beta;-Caroten-9-One/(-)-Methyl Jasmonate | -20.97 | -12.99 | -0.52 |
|  |  | 6-(4-Methyl-2-Oxopentyl)-4-Hydroxy-2-Pyrone | -0.17 | 10.74 | -0.13 |
|  |  | All-<I>Trans</I>-Hexaprenyl Diphosphate | -1.57 | -1.17 | -0.45 |
|  |  | Allocryptopine | -12.26 | -0.34 | 5.01 |
|  |  | Allosamidin | -2.35 | -1.58 | -0.85 |
|  |  | Berbamunine/Guattegaumerine | -1.79 | -1.20 | -0.60 |
|  |  | Bisdemethoxycurcumin | 19.84 | 12.95 | -0.21 |
|  |  | Carlactone | 1.30 | 0.76 | -17.64 |
|  |  | Decaprenyl Diphosphate | -0.70 | -1.28 | -0.94 |
|  |  | Deferoxamine Mesylate* | 18.54 | -0.40 | -0.15 |
|  |  | D-Erythro-Imidazole-Glycerol-Phosphate | -1.13 | -0.09 | -0.45 |
|  |  | D-Glucosamine 1-Phosphate | -17.08 | -5.78 | -11.57 |
|  |  | Dihydrocoumarin/4-Coumaraldehyde/1-Phenylpropane-1,2-Dione | -19.81 | -1.66 | -1.09 |
|  |  | Dihydrosanguinarine | 22.50 | 21.46 | 21.10 |
|  |  | Dimethylallyl Diphosphate/Isopentenyl Diphosphate | 16.70 | 16.65 | 16.62 |
|  |  | Dump/2'-Deoxyuridine 3'-Monophosphate | 16.31 | 16.18 | 2.48 |
|  |  | Epoxypheophorbide <I>A</I> | -1.60 | -1.22 | -0.49 |
|  |  | Fluorofumarate | 19.15 | 18.87 | 18.17 |
|  |  | Galactopinitol/D-Galactosylononitol | -17.52 | -11.55 | -0.11 |
|  |  | Gulonate/Glucoronate/Galacturonate | 3.42 | 21.07 | 17.52 |
|  |  | Imidazole Acetol-Phosphate | -1.23 | -2.02 | -1.43 |
|  |  | Inosine/Abscisate/4-Prenylphlorisobutyrophenone/Dihydroxyphaseic Acid | -1.85 | -1.54 | -0.27 |
|  |  | L-Dehydro-Ascorbate | -17.54 | 3.57 | 3.47 |
|  |  | Loganin | -18.78 | -0.56 | -0.43 |
|  |  | L-Phosphinothricin | 5.51 | 4.21 | 3.54 |
|  |  | Maltotriose/Raffinose/Kestotriose/Fagopyritol | -11.59 | -3.26 | 5.51 |
|  |  | Methoxyanigorufone | -1.91 | -1.58 | -0.28 |
|  |  | Methylphoracetophenone/(R)-3-(4-Hydroxyphenyl)Lactate | 1.67 | 0.36 | -0.34 |
|  |  | Methylsuccinate/Glutarate | -19.15 | -2.31 | -1.08 |
|  |  | Octyl &Beta;-D-Glucopyranoside* | -20.09 | -20.09 | -0.03 |
|  |  | Pamidronate | 17.40 | 10.99 | -0.21 |
|  |  | P-Chlorophenylalanine | -16.67 | -16.71 | -5.52 |
|  |  | Phenylarsine Oxide* | 14.01 | 13.97 | 13.94 |
|  |  | Pheophorbide <I>A</I> | -1.81 | -1.33 | -0.46 |
|  |  | Pheophorbide <I>B</I> | -17.19 | -4.72 | 1.76 |
|  |  | Pimelate | 5.02 | 3.68 | 2.95 |
|  |  | Plumbagin | -20.74 | -0.64 | 0.04 |
|  |  | Prostaglandin | -3.59 | -2.22 | -1.26 |
|  |  | Protochlorophyll A | -14.43 | -14.52 | -14.54 |
|  |  | Robustaquinone B/<I>Trans</I>-Cinnamoyl-&Beta;-D-Glucoside | 22.39 | 21.37 | 21.02 |
|  |  | Robustaquinone G | 19.97 | -0.06 | -0.29 |
|  |  | Rosmarinate/<I>S</I>-(Hydroxymethyl)Glutathione | 19.72 | 18.62 | 5.81 |
|  |  | Salicylate/Hydroxybenzoate | 16.44 | 10.73 | -0.21 |
|  |  | Sanguinarine | 12.72 | 11.70 | 11.35 |
|  |  | Suberate/6-Diazo-5-Oxonorleucine | -0.08 | 5.74 | -0.21 |
|  |  | Thenoyl Trifluoro Acetone* | -5.58 | 5.62 | 10.67 |
|  |  | Tricaffeoyl Spermidine | -17.79 | -17.85 | -17.87 |
|  |  | Triethanolamine | -17.62 | -17.90 | -1.06 |
|  |  | Triferuloyl Spermidine | -13.01 | -13.29 | 5.52 |
|  |  | Uridine* | -18.43 | -18.36 | -0.64 |

Supplementary Table 5: Primers employed in this study and their main features.

| Primer name | Sequenza (5’-3’) | Ta^1^ | AL^2^ |
| --- | --- | --- | --- |
| ObNTR1.4 Fw | GCCTGCGAAGCAATCAACTC | 55 | 122 |
| ObNTR1.4 Rv | ATCATCGAACTGGTCGGCTC | 55 | 122 |
| Ob-GPDH Fw | AGGCTAGAGAAGGAGGCCAC | 55 | 113 |
| Ob-GPDH Rv | TCGGTGGAGACCACATCGTC | 55 | 113 |
| ObNTR1.1 Fw | GCATCGGAGCTTCATCAGGA | 55 | 139 |
| ObNTR1.1 Rv | TGTTCGGGTTGAACTGGTCC | 55 | 139 |

^1^ Ta: Annealing temperature

^2^ AL: Amplicon lenght (bp)
