# Supplementary figures and images for: Metabolic Insights into the Anion-Anion Antagonism in Sweet Basil: Effects of Different Nitrate/Chloride Ratios in the Nutrient Solution
Source: Int J Mol Sci. 2020 Apr 3;21(7):2482. doi: 10.3390/ijms21072482 (PMC7177776; doi:10.3390/ijms21072482)

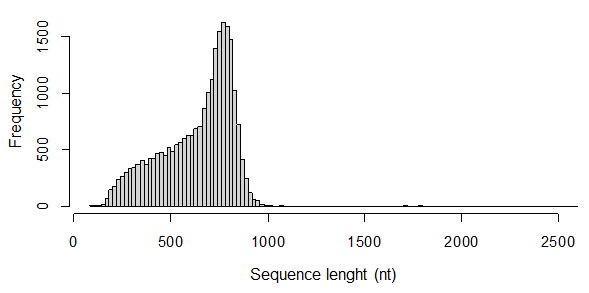

Supplement: Supplementary file 1 [file ijms-21-02482-s001.zip › Supplementary Files/Supplementary Figure 1.tif]

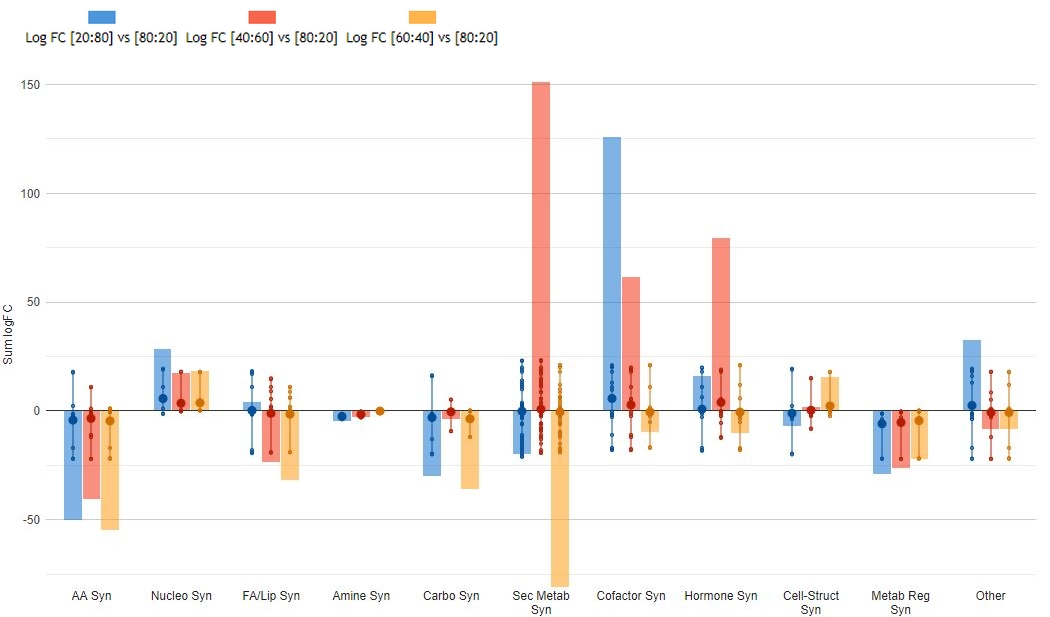

Supplement: Supplementary file 1 [file ijms-21-02482-s001.zip › Supplementary Files/Supplementary Figure 2.jpg]

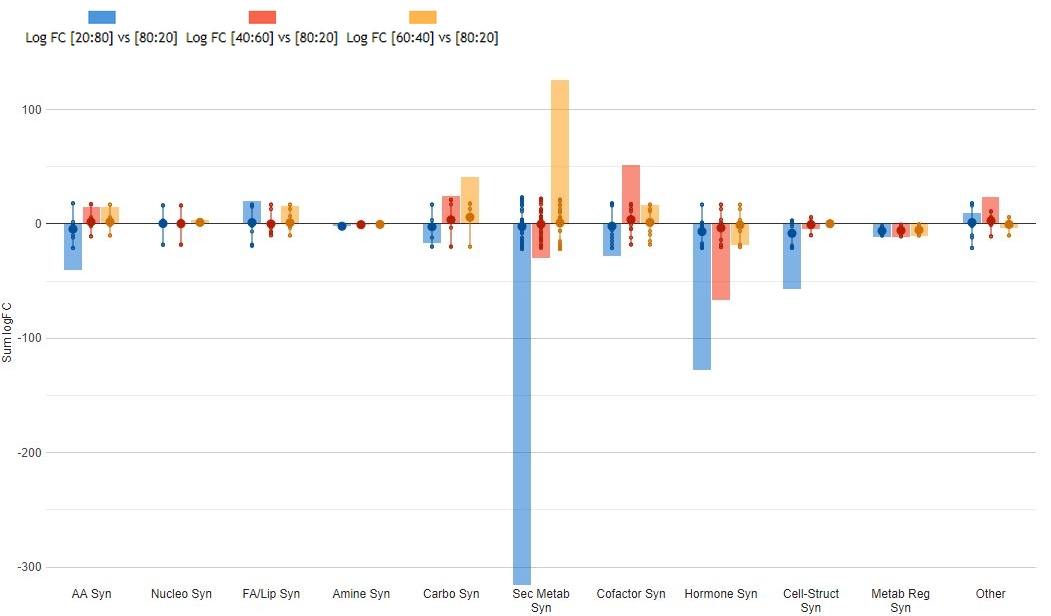

Supplement: Supplementary file 1 [file ijms-21-02482-s001.zip › Supplementary Files/Supplementary Figure 3.jpg]

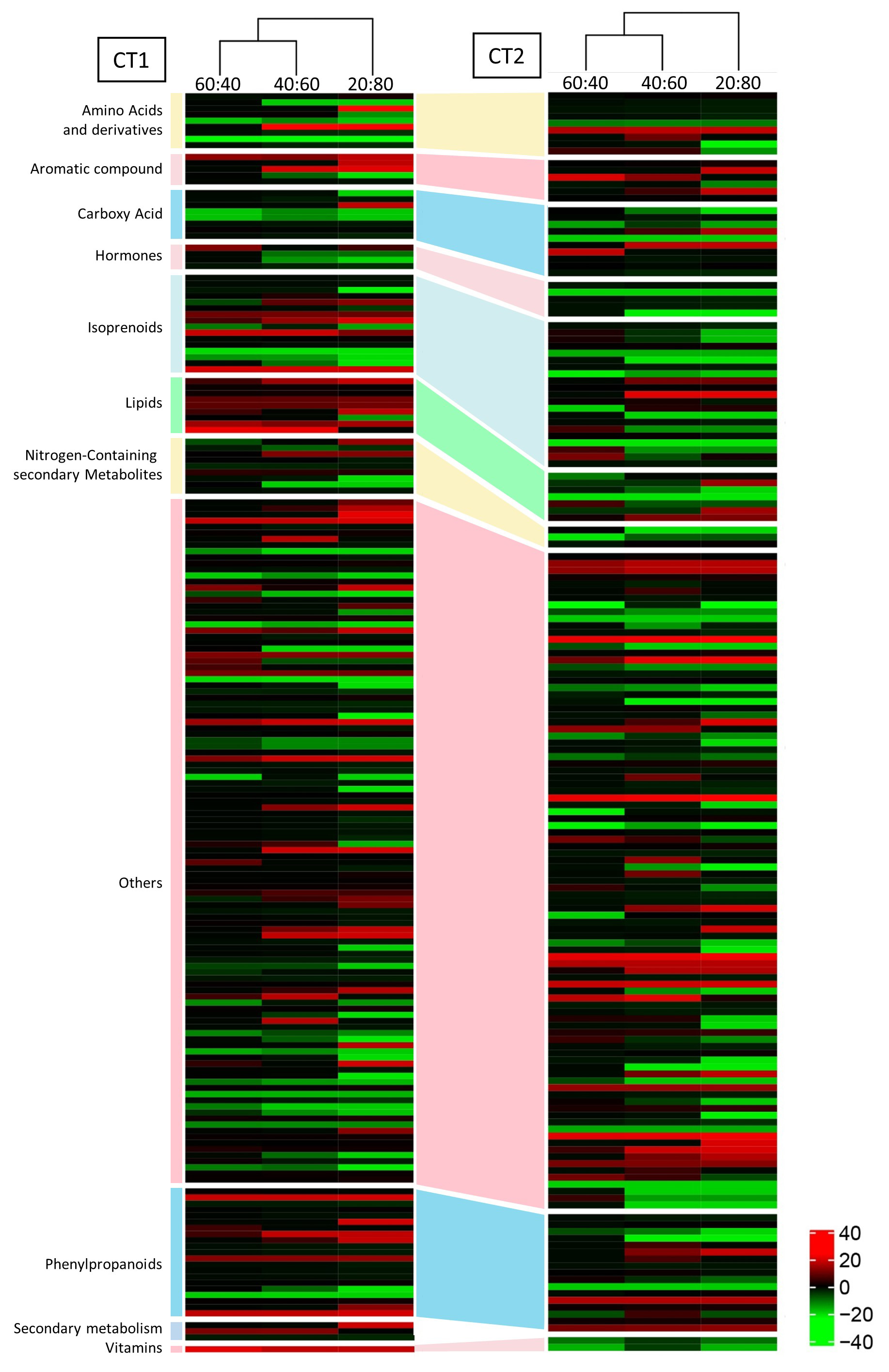

Supplement: Supplementary file 1 [file ijms-21-02482-s001.zip › Supplementary Files/Supplementary Figure 4.jpg]
